# Isolation and proteomic analysis of intracellular vesicles from the potato late blight pathogen *Phytophthora infestans*

**DOI:** 10.1038/s41598-026-37161-2

**Published:** 2026-01-25

**Authors:** Jasmine Pham, Stephen C. Whisson, Charlotte H. Hurst, Sean Chapman, Paul R. J. Birch

**Affiliations:** 1 Division of Molecular Cell and Developmental Biology, School of Life Sciences, University of Dundee, Dundee, DD2 5DA UK; 2 Division of Plant Sciences, University of Dundee, at The James Hutton Institute, Errol Rd, Invergowrie, Dundee, DD2 5DA UK; 3 Department of Cell and Molecular Sciences, The James Hutton Institute, Invergowrie, Dundee, DD2 5DA UK; 4 Medical Research Council Protein Phosphorylation and Ubiquitylation Unit, School of Life Sciences, University of Dundee, Dundee, DD2 5DA UK

**Keywords:** *Phytophthora infestans*, Effector proteins, Protein secretion, Intracellular vesicles, Gradient ultracentrifugation, Data-independent acquisition (DIA) mass spectrometry, Biotechnology, Microbiology, Molecular biology, Plant sciences

## Abstract

The oomycete *Phytophthora infestans* is a filamentous plant pathogen and the causal agent of potato late blight resulting in annual crop losses of billions of dollars worldwide. Key to the success of this pathogen are the effector proteins it secretes during infection, whose functions include breaching the plant cell wall and suppression/evasion of plant immunity. Currently, little is known regarding the intracellular trafficking of effectors enroute to secretion. Here, we developed a robust density gradient ultracentrifugation method to isolate and separate diverse populations of intracellular vesicles from *P. infestans* based on buoyancy for the purpose of identifying vesicle-associated proteins by DIA-mass spectrometry. Gene Ontology Enrichment Analysis of buoyant fractions revealed enrichment for proteins associated with membranes and vesicle trafficking. Buoyant fractions were also enriched in RXLR effectors, carbohydrate-active enzymes, and signal peptide containing secretory proteins, all representing potential vesicle cargo. Recently identified *P. infestans* extracellular vesicle markers were also present. Unravelling how effector proteins are trafficked for secretion is a critical step in developing robust strategies for combating potato late blight disease; the proteomics dataset and method presented here are valuable resources from which potential biomarkers for *P. infestans* vesicles can be identified for future studies towards this end.

**Supplementary Information:**

The online version contains supplementary material available at 10.1038/s41598-026-37161-2.

## Introduction

*Phytophthora infestans* is a major pathogen of potato and tomato, causing annual losses amounting to more than €1 billion within the European Union alone^1^. *P. infestans* is notoriously difficult to control in the field, with current control methods based on costly fungicides, some with unknown modes of action. Attempts to breed crops with long-lasting resistance have generally been unsuccessful^1,2^. Therefore, unravelling how *P. infestans* invades the host plant is a critical for developing robust strategies to combat this plant pathogen.

*P. infestans* is an oomycete belonging to the kingdom Stramenopila, and although resembling fungi in appearance, the oomycetes are distinguished by their coenocytic hyphae lacking septa, and the presence of cellulose rather than chitin in their cell walls^3^. During infection, *P. infestans* develops haustoria, finger-like projections that form intimate interactions with the plant cell membrane. It is at this pathogen-plant interface that *P. infestans *secretes and delivers a range of proteins, small molecules and RNAs. Their functions range from assisting evasion of plant immunity, to physically breaching plant tissue, and nutrient uptake (reviewed in Boevink et al.^4^).

Amongst the proteins secreted during infection by *P. infestans*, effector proteins have been the focus of intense research and are divided into two broad classes: apoplastic (extracellular) and cytoplasmic (intracellular) effectors. Apoplastic effectors, which act within the apoplastic space outside the plant cell, often function as hydrolytic/cell wall degrading enzymes, such as pectin esterases^5^. Other apoplastic effector functions include nutrient scavenging (as is the case for elicitins which are reported to facilitate sterol and lipid acquisition^6^; various proteases (e.g., aspartic, cysteine and metalloproteases) (reviewed in Saraiva et al.^7^); and inhibitors of plant hydrolases and proteases^8–10^ secreted in defence against *P. infestans*.

In contrast, cytoplasmic effectors are translocated into the plant cell. The most well studied in *P. infestans* are the RxLR effectors, with 563 encoded within the genome^11^. These effectors are characterised by the presence of an Arg-x-Leu-Arg motif (with ‘x’ denoting any amino acid) downstream of a signal peptide near the N terminus. RxLR effector protein function arises from their variable C-termini and accounts for their wide-ranging functions, which include interference/modulation of host signalling, protein regulation, transcription, RNA trafficking and processing, and cellular trafficking^12^.

Given the importance of proteins secreted by *P. infestans* in modulating the outcome of infection and disease, much attention has focussed on the fate and function of these proteins once secreted. However, the intracellular trafficking pathways enroute to extracellular secretion remain understudied. The ability to track the intracellular pathways of effectors would aid in developing novel targeted control methods to disrupt effector secretion and inhibit infection. Achieving this will require the identification of markers for intracellular compartments and vesicles through, and by, which these proteins are trafficked.

A bottleneck for studying protein secretion in *P. infestans*, and indeed other filamentous plant pathogens, is the lack of well-characterised vesicle markers such as those found in mammalian systems^13,14^. Notably, tetraspanins, a group of well conserved multi-pass transmembrane proteins routinely used as markers for vesicles in mammalian research^13^, have only recently been identified in *Phytophthora sojae* extracellular vesicles (EVs)^15^. In *P. infestans*, two transmembrane MARVEL domain proteins have emerged as potential markers for EVs associated with RXLR effectors^16^.

Despite the progress in identifying EV-associated proteins, little research has been carried out to identify intracellular vesicle proteins. Here, we present a robust method for isolating intracellular vesicles from *P. infestans* to identify potential marker and cargo proteins by mass spectrometry. Differential centrifugation is a common strategy for isolating vesicles, utilising lower speed centrifugations to remove cellular debris followed by high-speed ultracentrifugation to pellet vesicles, which are then resuspended for further separation and/or analysis. However, pelleting vesicles in this way can cause vesicles to rupture, deform and aggregate with each other or with contaminating proteins^17,18^. To avoid this effect, cushioned ultracentrifugation has been employed to replace the final high-speed ultracentrifugation step, whereby vesicles are concentrated on top of a high-density gradient medium, preventing pellet formation^19^.

Following on from vesicle concentration, gradient ultracentrifugation is commonly used to separate different classes of vesicles based on physical properties. In this method, a gradient of high to low densities formed of a gradient medium such as sucrose or iodixanol is either overlaid/top-loaded or underlaid/bottom-loaded with the sample. In the case of bottom-loaded gradients, particles are separated based solely on buoyancy^18,20^.

Although differential centrifugation, cushioned ultracentrifugation and bottom-loaded gradient ultracentrifugation are individually well established techniques, to our knowledge, they have not been combined together for the isolation of vesicles from fungal or oomycetes plant pathogens (reviewed in Palatinus et al.^21^). Combining these techniques, we isolated intracellular *P. infestans* vesicles based on buoyancy and identified associated proteins by state-of-the-art data-independent acquisition (DIA) mass spectrometry to generate a robust dataset of intracellular vesicle-associated proteins.

This dataset represents the first community resource to identify markers for both intracellular compartment vesicles and cargo proteins from *Phytophthora infestans* for future studies. The robust method presented here can also be adapted in future studies to investigate specific intracellular vesicle populations in more detail. This resource will enable dissection of the intracellular trafficking pathways of effector proteins, with a view to identifying novel methods for controlling this devastating potato pathogen.

## Results

### Generation of a dual expression vector enables visualisation and tracking of fluorescently-tagged secreted proteins

In the absence of an available vesicle marker for *P. infestans*, we instead used two effector proteins as markers for secretory proteins/vesicle cargo: PITG_04314, a representative of the RXLR effectors, and the pectin esterase PITG_01029, a host cell wall modifying enzyme within the apoplastic effector category^11^.

**Fig. 1:**
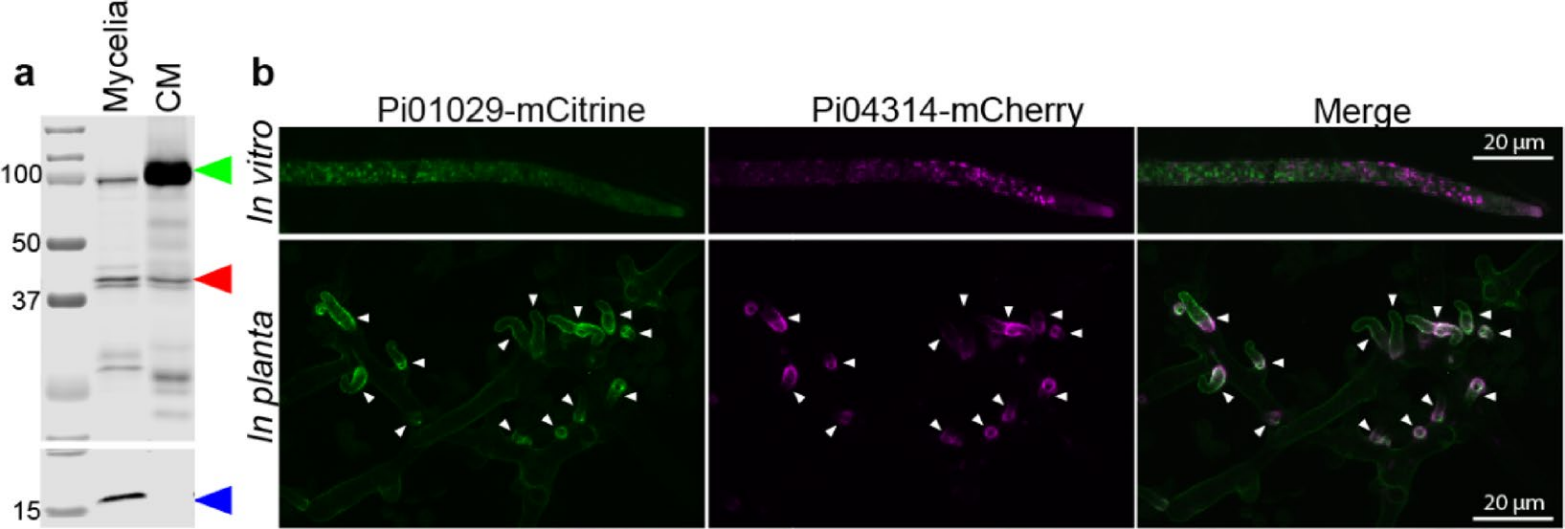
Simultaneous localisation of PITG_04314-mCherry and PITG_01029-mCitrine expressed from a dual expression vector in *P. infestans.* (**a**) Western blot analysis of *in vitro* grown mycelia and the corresponding conditioned media (CM). Green left-pointing triangle: PITG_01029-mCitrine, Red left-pointing triangle: PITG_04314-mCherry, Blue left-pointing triangle: histone. Sizes of marker proteins (kDa) are indicated. Western blots have been cropped for clarity. Original blot image is presented in Supplementary Figure S2. (**b**) Confocal microscopy of mycelia grown in vitro (upper panel) and *in planta* (lower panel). White arrowheads indicate location of haustoria. Scale bars: 20 μm.

To track the cellular location of these two effector proteins, a dual expression vector (pDual_mCherry_mCitrine, Supplementary Fig. S1) was constructed and used to simultaneously express PITG_04314-mCherry with PITG_01029-mCitrine in *P. infestans*. Mycelia of the dual transformant was grown in vitro in liquid culture and western blot analysis of proteins extracted from mycelia and conditioned medium confirmed both tagged proteins were expressed and secreted (Fig. 1a). PITG_01029-mCitrine ran to an approximate size of 100 kDa, larger than the expected 61.5–63 kDa, and PITG_04314-mCherry ran as a doublet in the expected size range of 42–44.5 kDa. A deglycosylation assay confirmed the size shift in PITG_01029-mCitrine was due to posttranslational modification, whereas PITG_04314-mCherry remained unaltered (Supplementary Fig. S2).This suggests presence of the doublet for PITG_04314-mCherry was not due to glycosylation state and may instead be due to proteolytic processing of the protein^22^.

Confocal microscopy of mycelia grown in vitro confirmed the correct expression and folding of the fluorescent tags, with both tagged proteins localising to punctate structures (Fig. 1b, upper panel). Confocal imaging of the transformant *in planta* during infection of *Nicotiana benthamiana* leaves shows both PITG_04314-mCherry and PITG_01029-mCitrine accumulating at the haustoria (Fig. 1b, lower panel).

### Buoyant density ultracentrifugation facilitates robust and reproducible isolation of *P. infestans* intracellular vesicles

**Fig. 2:**
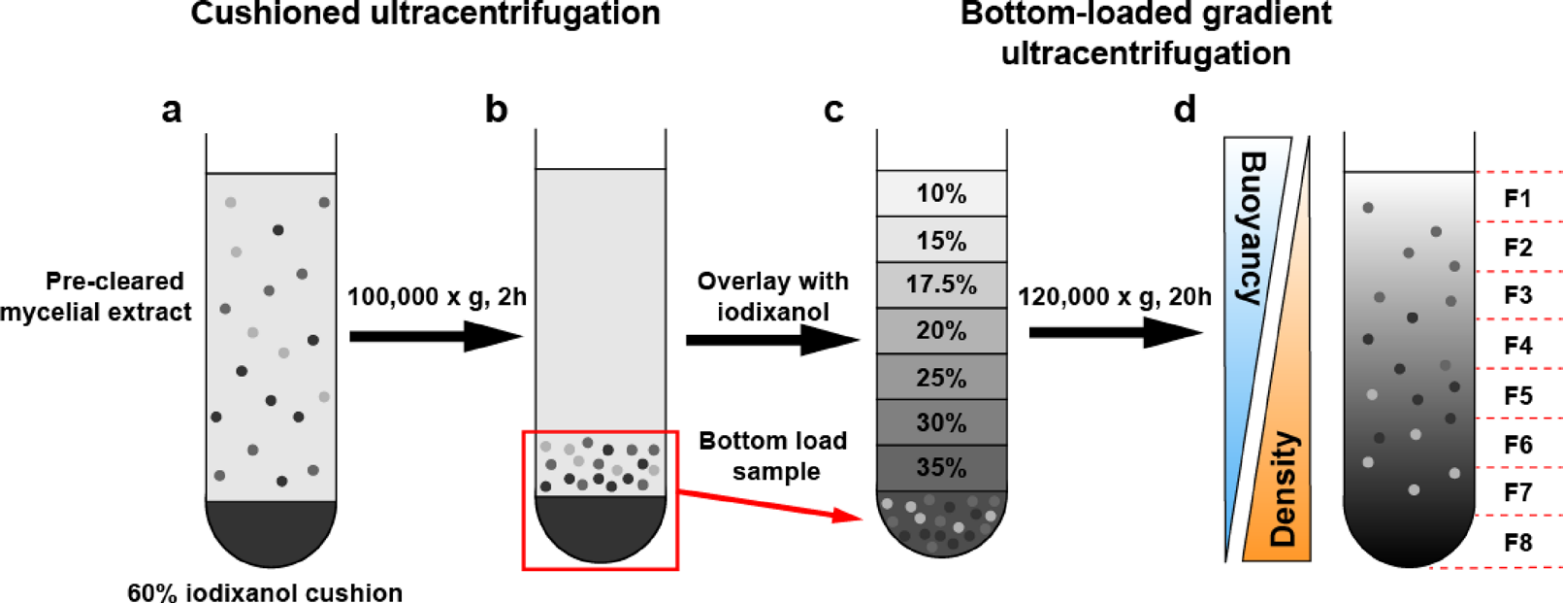
Overview of cushioned-bottom loaded iodixanol gradient ultracentrifugation method used to isolate *P. infestans* intracellular vesicles based on buoyancy. (**a**) Pre-cleared mycelial extract containing intracellular vesicles in isolation buffer were loaded on top of a 60% iodixanol cushion and centrifuged at 100,000 × g for 2 h. (**b**) After centrifugation, the vesicles concentrate above the cushion. (**c**) The iodixanol cushion and 1 ml above the cushion containing the concentrated vesicles were mixed, bottom loaded into a new tube, then overlayed with fractions of iodixanol with progressively lower densities to form a discontinuous gradient. (**d**) After centrifugation at 120,000 × g for 20 h, the gradient becomes continuous and vesicles migrate up the gradient based on buoyancy, with denser material remaining near the bottom/denser fractions. Eight fractions were taken starting from the top of the tube to the bottom for analysis (fractions F1-F8).

As both PITG_04314-mCherry and PITG_01029-mCitrine were confirmed to be secreted from mycelia, we set out to isolate the intracellular vesicles in which these proteins are trafficked along the secretory pathway. Using the dual transformant, we isolated intracellular vesicles from *in vitro* grown mycelia, using cushioned bottom-loaded density gradient ultracentrifugation (Fig. 2). With this technique, vesicles are concentrated onto a high density iodixanol cushion without pellet formation, which increases recovery and preserves the integrity and morphology of the vesicles^19^. The concentrated vesicles were then bottom-loaded and overlaid with fractions of iodixanol of progressively lower densities. Iodixanol was chosen as the gradient medium due to its low viscosity and iso-osmotic properties which better preserve vesicle size and density, resulting in reproducible separation of vesicles compared to gradient mediums such as sucrose^23^. During ultracentrifugation, a gradient of continuous densities is formed, and the vesicles migrate up through the gradient to a point at which their density matches that of the surrounding gradient medium (isopycnic position), separating the vesicles based solely on buoyancy. As lipids form the main component of vesicle membranes and have a lower density than proteins (1 g/cm^3^ versus 1.3–1.5 g/cm^3^, respectively), protein aggregates tend to accumulate away from the vesicles towards fractions of higher density, along with heavier cellular debris^20,24^.

**Fig. 3:**
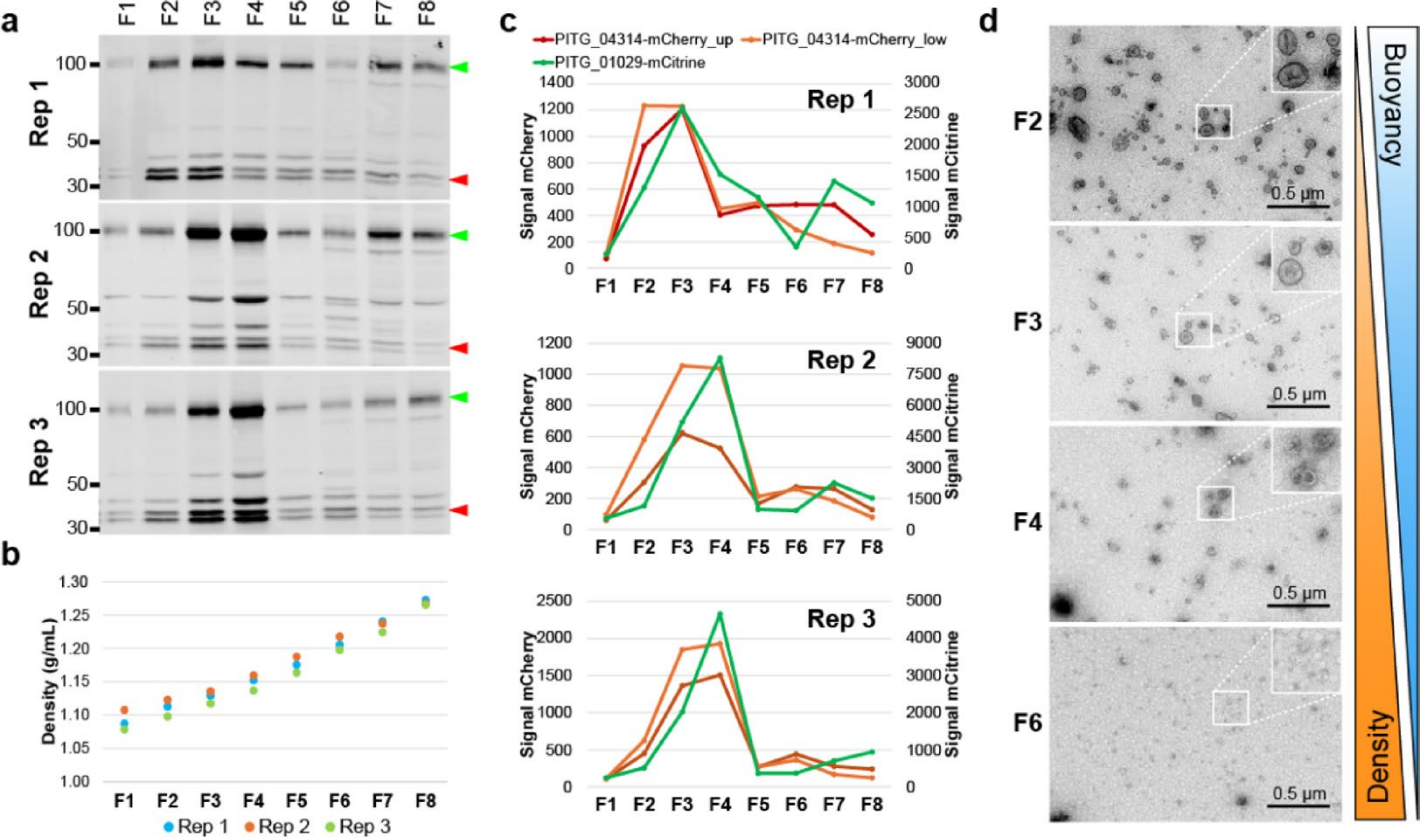
PITG_04314-mCherry and PITG_01029-mCitrine migrate to fractions of lower density within the gradient, coinciding with the presence of vesicle-like structures. (**a**) Western blot of fractions taken from three independent gradients. Green left-pointing triangle: PITG_01029-mCitrine, red left-pointing triangle : PITG_04314-mCherry. Sizes of marker proteins (kDa) are indicated. Western blots have been cropped for clarity. Original blot images are presented in Supplementary Figure S3. (**b**) Density measurements calculated from OD 340 nm readings of the fractions shown in Fig. 3a. (**c**) Quantification of band intensities for PITG_04314-mCherry and PITG_01029-mCitrine shown in Fig. 3a. PITG_04314-mCherry appears as a doublet; intensity of the upper (‘PITG_04314-mCherry_up’) and lower (‘PITG_04314-mCherry_low’) were quantified separately. (**d**) Transmission electron microscopy (TEM) images of selected fractions. Areas enclosed by small white boxes are magnified in the top righthand corner of the image. Scale bars: 0.5 μm.

Replicate experiments revealed the gradients to be reproducible in terms of floatation of PITG_04314-mCherry and PITG_01029-mCitrine up the gradient and density measurements of the fractions. PITG_04314-mCherry and PITG_01029-mCitrine consistently migrated up to regions of lower density, predominantly fractions F2, F3 and F4 (Fig. 3a, c), corresponding to average densities of 1.111 ± 0.013 g/mL, 1.128 ± 0.009 g/mL and 1.150 ± 0.012 g/mL, respectively (Fig. 3b). These densities were within the range observed for EVs isolated from the fungal phytopathogen *Colletotrichum higginsianum* isolated by a similar approach^25^. Total protein staining showed migration of proteins up the gradient, with a clear distinction in protein profiles between the lower density fractions F1-F4 and the higher density fractions F5-F8 (Supplementary Fig. S3). Transmission electron microscopy (TEM) was used to image fractions, a common method used to visualise the presence and purity of vesicles. With this technique, vesicles commonly appear collapsed/cup-shaped due to the process of fixing and drying of the sample required for imaging^15,25,26^. Imaging of fractions F2, F3, and F4 showed the presence of vesicular structures, whereas the higher density fraction F6, containing little PITG_04314-mCherry and PITG_01029-mCitrine, showed a near absence of vesicle-like structures (Fig. 3d).

### Disruption of vesicle integrity prevents differential protein migration within the gradient

Addition of detergent such as Triton X-100 to the sample prior to bottom-loading of the gradient is expected to disrupt membrane-bound structures such as vesicles, causing release of protein cargo and disrupting floatation. This strategy is commonly used to confirm a protein of interest as vesicle cargo, with TEM used to confirm disruption of vesicles^14^.

**Fig. 4:**
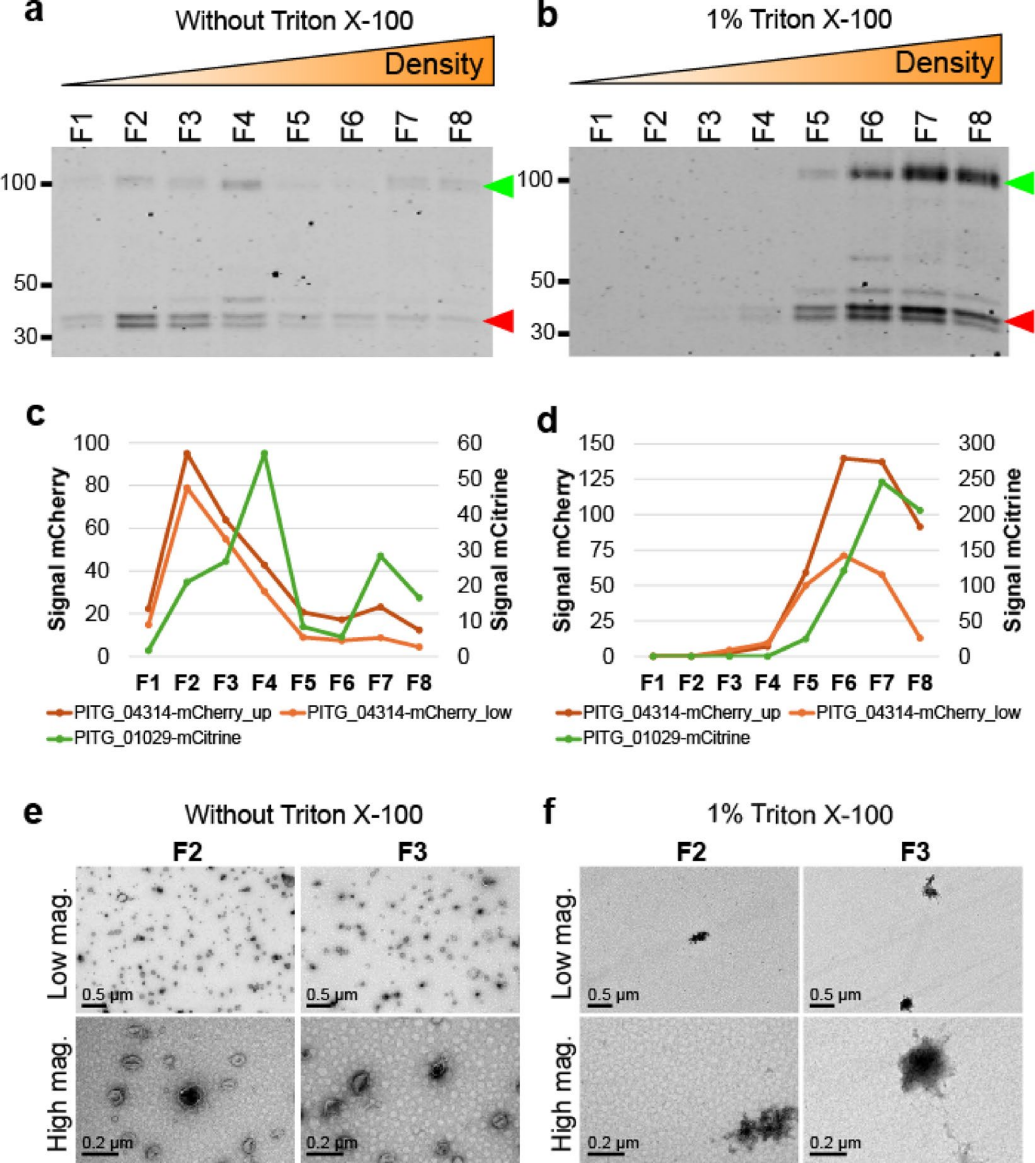
Preincubation of samples with 1% Triton X-100 prior to bottom-loading of the gradient prevents migration of PITG_04314-mCherry and PITG_01029-mCitrine up the gradient, coinciding with the absence of vesicle-like structures. (**a**) Western blot showing position of PITG_04314-mCherry and PITG_01029-mCitrine within the fractions of a gradient in the absence of Triton X-100. Sizes of marker proteins (kDa) are indicated. Green left-pointing triangle: PITG_01029-mCitrine, red left-pointing triangle: PITG_04314-mCherry. Western blots have been cropped for clarity. Original blot images are presented in Supplementary Figure S4. (**b**) Western blot showing position of PITG_04314-mCherry and PITG_01029-mCitrine within a gradient on pre-incubation of the sample with 1% Triton X-100 prior to bottom-loading. Sizes of marker proteins (kDa) are indicated. Green left-pointing triangle: PITG_01029-mCitrine, red left-pointing triangle: PITG_04314-mCherry. (**c**) Quantification of band intensities for PITG_04314-mCherry and PITG_01029-mCitrine shown in Fig. 4a (without Triton X-100). (**d**) Quantification of band intensities for PITG_04314-mCherry and PITG_01029-mCitrine shown in Fig. 4b (with 1% Triton X-100). (**e**) Transmission electron microscopy (TEM) images of fractions F2 and F3 in the absence of Triton X-100 (Fig. 4e) and in the presence of 1% Triton X-100 (Fig. 4f). Low magnification scale bars: 0.5 μm, High magnification scale bars: 0.2 μm.

Without Triton X-100 pretreatment, PITG_04314-mCherry and PITG_01029-mCitrine migrated predominantly to fractions F2, F3 and F4 as expected (Fig. 4a, c). On the other hand, sample pretreated with 1% Triton X-100 run in parallel showed a measurable shift in the migration of PITG_04314-mCherry and PITG_01029-mCitrine, with accumulation towards the bottom/denser portions of the gradient (Fig. 4b, d). Pretreatment with Triton X-100 affected protein migration within the gradient in general, as total protein staining of western blots showed increased accumulation of total protein in fractions F5-F8 with the absence of detectable protein staining in fractions F1-F4 (Supplementary Fig. S4). This suggests that Triton X-100 disrupts intracellular vesicles in general. TEM of fractions F2 and F3 in the untreated sample revealed the presence of vesicular structures, which were absent with Triton X-100 pre-treatment (Fig. 4e, f).

Since Triton X-100 has previously been shown to disrupt membranes whilst leaving protein aggregates intact^27^, the results suggest that PITG-04314-mCherry and PITG-01029-mCitrine are likely trafficked within the cell in vesicles and that our protocol enables isolation of these vesicles whilst preserving their integrity.

### Presence of vesicular structures in buoyant fractions correlates with differences in protein abundance profiles

As PITG_04314-mCherry and PITG_01029-mCitrine migrated predominantly to the lower density fractions F2, F3 and F4 (collectively referred to as the ‘buoyant fractions’ from hereon), these fractions were analysed to identify proteins associated with *P. infestans* intracellular vesicles. Since the high-density fraction F6 represents the first fraction the vesicles can float up through (fractions F8 and F7 span the sample loading zone) and was found to be devoid of vesicle-like structures, we included this fraction to account for potential contaminants/proteins not associated with intact vesicles.

Across the four fractions analysed, a total of 6,685 *P. infestans* protein groups were detected (i.e., present in at least two samples within the entire experiment). To account for variations in protein abundance between samples, raw intensities of each sample were Log_2_ transformed and normalised to the sample median intensity before statistical analysis (Supplementary Fig. S5, Supplementary Data 1).

**Fig. 5:**
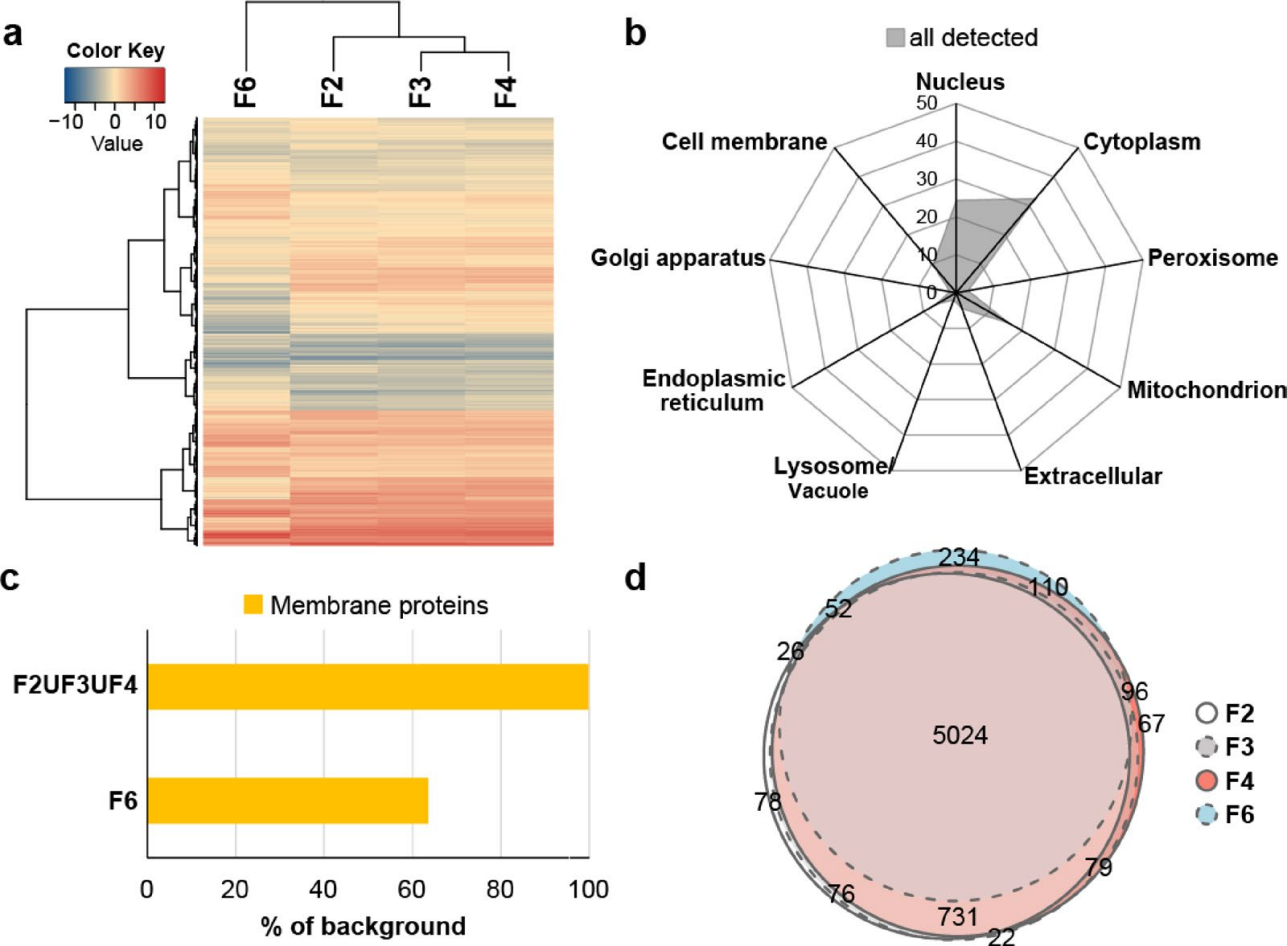
Overview of Data-Independent Acquisition mass spectrometry data of fractions F2, F3, F4 and F6 from three biological replicate experiments. (**a**) Heatmap displaying hierarchical clustering of fractions based on similarities in protein intensity for the 6,685 protein groups detected by mass spectrometry run in Data-Independent Acquisition (DIA) mode for each fraction. (**b**) Radar chart showing DeepLoc predicted cellular association of the detected proteins as a percentage of total proteins detected. (**c**) Number of membrane proteins detected in at least one buoyant fraction (F2ՍF3ՍF4) and F6 as a percentage of the 1,626 predicted membrane proteins detected in the entire dataset. (**d**) Euler diagram showing overlap in proteins detected (present in at least two reps) for fractions F2, F3, F4 and F6. The overlapping 5,024 proteins present in all four fractions were taken forward for comparison of protein abundance between fractions.

Hierarchical clustering based on protein abundance profiles across fractions revealed the buoyant fractions to be more similar to each other than to the denser fraction F6 (Fig. 5a).

Prediction of cellular localisation of the detected proteins revealed the most abundant proteins to be associated with the cytoplasm (32.5%), nucleus (24.4%) and mitochondrion (15.9%) (Fig. 5b). Lower abundance proteins were associated with cell membrane (9.5%), endoplasmic reticulum (6.3%), extracellular (4.5%), peroxisome (3.2%), lysosome/vacuole (2.0%) and Golgi apparatus (1.7%). Classification of proteins as either membrane or soluble revealed all but three (PITG_13657, PITG_06609 and PITG_15810) of the 1,626 detected membrane proteins could be found in at least one of the buoyant fractions (Fig. 5c). In contrast, only 594 of the total detected membrane proteins (36.5%) were detected in F6 (Fig. 5c).

Within individual fractions, the number of proteins present (i.e., identified in at least two biological replicates of the fraction) were 6,057, 6,078, 6,199 and 5,592 proteins for fractions F2, F3, F4 and F6 (respectively), with an overlap of 5,024, suggesting high similarity regarding the identity of proteins present within each fraction (Fig. 5d). These observations suggest differences in protein abundance, rather than presence or absence, as the main factor determining differences between buoyant and dense fractions. Indeed, analysis of protein presence/absence revealed 283 proteins that are exclusively associated with all three buoyant fractions, and only three proteins exclusively found in F6 (Supplementary Fig. S6).

**Fig. 6:**
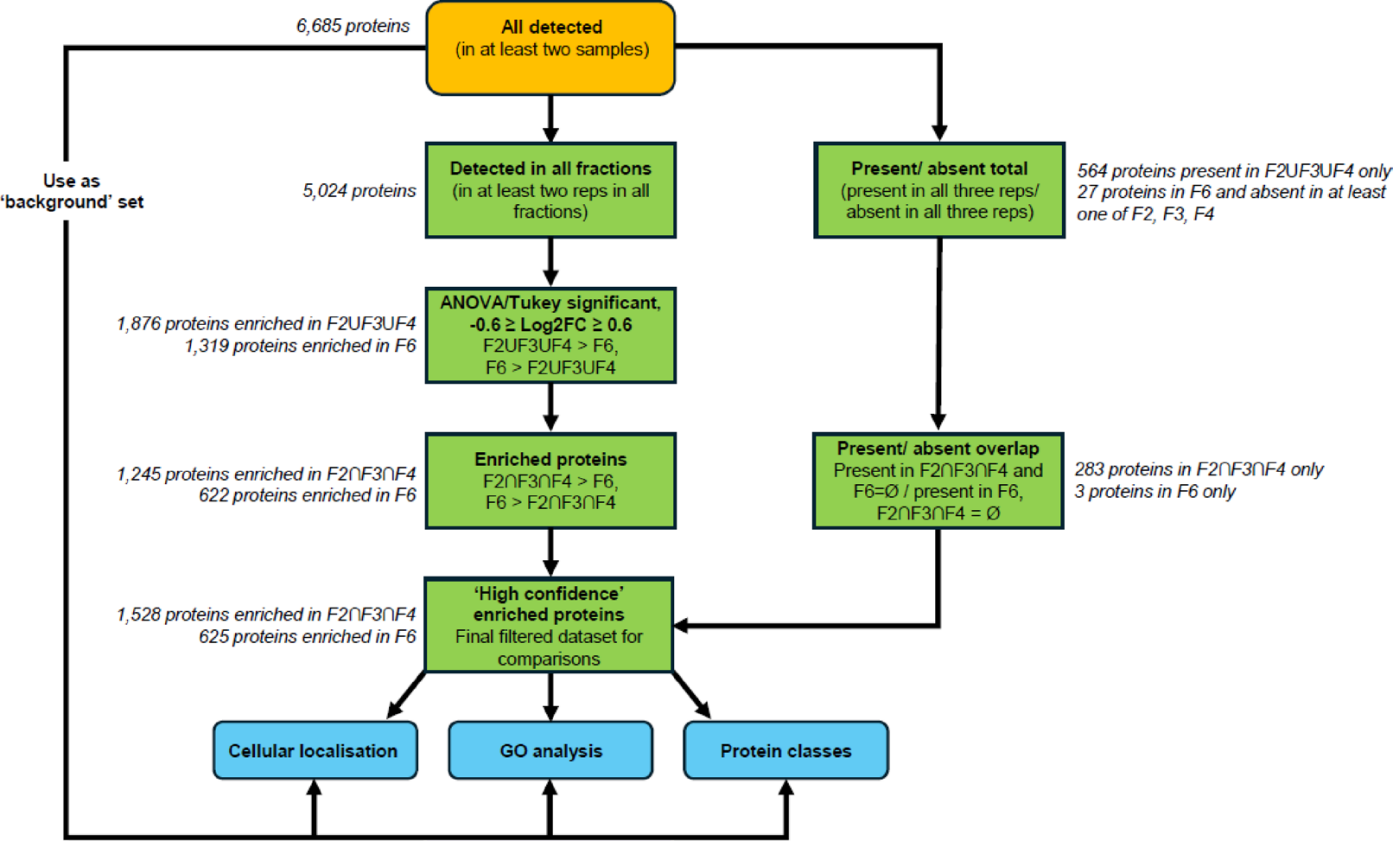
Schematic of data analysis workflow. A set of high confidence proteins for enrichment analysis were derived from filtering for proteins based on presence/absence and Log_2_ fold-change (Log_2_FC). ‘F2ՍF3ՍF4’: set of proteins representing the union of proteins detected in F2, F3 and F4 (i.e., present in at least two reps for at least one of the fractions), ‘F2∩F3∩F4’: set of proteins representing the intersect of proteins detected in F2, F3 and F4 (i.e., present in at least two reps for all fractions), ‘F6 = Ø’: where F6 is the empty set in relation to presence/absence, ‘F2∩F3∩F4 = Ø’: where F2∩F3∩F4 is the empty set in relation to presence/absence.

Given that we were interested in proteins enriched in the buoyant fractions associated with intracellular vesicles, enrichment analysis focussed on comparison of fractions F2, F3 and F4 versus the denser fraction F6, which was shown to be devoid of vesicles. The flowchart in Fig. 6 summarises the workflow used for protein enrichment analysis.

### Buoyant fractions F2, F3 and F4 are enriched for a common set of proteins

The 5,024 proteins common between all fractions were further analysed for differential abundance between fractions. One-way ANOVA (FDR 0.05, q-value < 0.05 with Tukey’s HSD test (FDR 0.05)) was performed to identify proteins which differed significantly between the buoyant fractions and fraction F6, returning a total of 3,320 proteins. Log_2_ fold-change (Log_2_FC) was calculated for each protein as the ratio of median normalised intensities for each buoyant fraction against fraction F6. The proteins were then further filtered based on a Log_2_FC cutoff of −0.6 ≥ Log_2_FC ≥ 0.6 to obtain the differentially abundant proteins within the fractions; those more abundant in at least one of the buoyant fractions (F2ՍF3ՍF4 > F6, Log_2_FC ≥ 0.6), of which there were 1,876, and those more abundant in the high density fraction F6 compared to at least one buoyant fraction (F6 > F2ՍF3ՍF4, Log_2_FC ≤ −0.6), of which there were 1,319.

Within the 1,876 proteins enriched in at least one of the buoyant fractions, 1,245 intersected or were more abundant in all three fractions over F6 (F2ՈF3ՈF4 > F6, Log_2_FC ≥ 0.6) (Supplementary Fig. S6). These 1,245 proteins were combined with the 283 proteins that were present in all three buoyant fractions and absent in F6 to give a set of 1,528 high confidence proteins potentially associated with intracellular vesicles.

Of the 1,319 proteins identified as more abundant in F6 over at least one buoyant fraction, 622 of these were enriched in F6 over all three buoyant fractions (F6 > F2ՈF3ՈF4, Log_2_FC ≤ −0.6) (Supplementary Fig. S6). These 622 proteins were combined with the three proteins present exclusively in F6 to give a set of 625 high confidence proteins enriched in fraction F6, representing potential contaminant proteins not associated with intracellular vesicles.

With the 1,528 and 625 high confidence proteins enriched in the buoyant fractions and F6, respectively (Supplementary Data 1), we proceeded to classify the types of proteins within these two datasets, in the context of the full background set of all 6,685 detected proteins.

### Buoyant fractions are enriched for membrane proteins and signal peptide containing secretory proteins

**Fig. 7:**
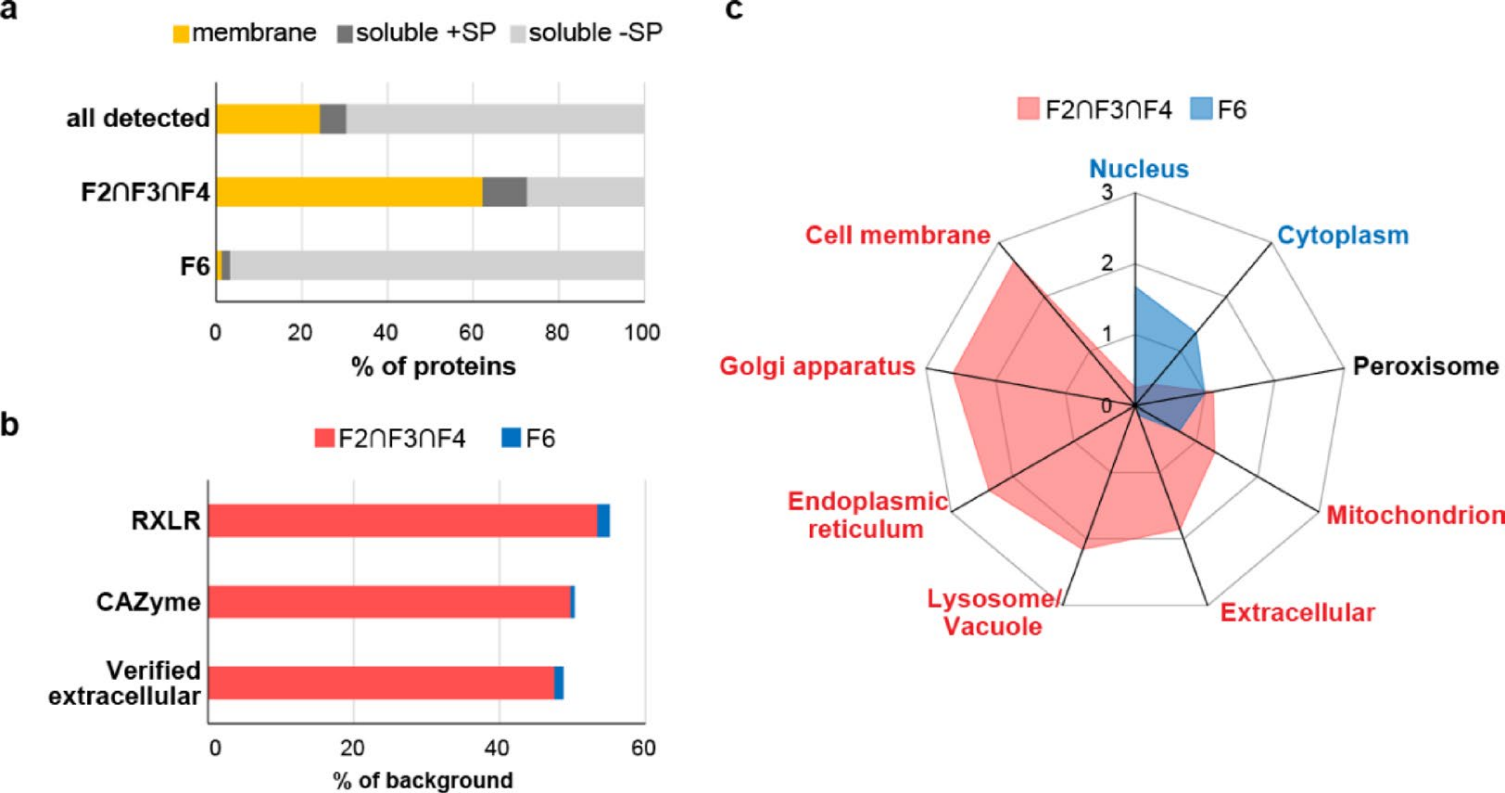
Classification and associated cellular localisation of proteins enriched in fractions F2, F3, F4 and F6. (**a**) Percentage of proteins predicted membrane associated or soluble found in all three buoyant fractions (F2∩F3∩F4), and within F6. The predicted soluble proteins were further subdivided into those with (‘soluble +SP’) and without (‘soluble -SP) a signal peptide. The proportion of each class of protein for the background set of all 6,685 detected proteins (‘all detected’) is also shown for comparison. (**b**) Number of RXLR and CAZymes associated with the buoyant fractions and with F6 shown as a percentage of the total number of these proteins in the background set of all detected proteins. The number of ‘verified extracellular’ proteins were determined by comparison to a list of proteins previously identified as secreted into the extracellular space by *P. infestans.* See main text for details. (**c**) Radar chart showing percentage of proteins associated with each cellular compartment (predicted by DeepLoc) as a ratio of the percentage found in the background set (fold-enrichment) for the buoyant fractions (F2∩F3∩F4, pink filled square) and F6 (blue filled square). Cellular localisation labelled in pink showed significant association with the buoyant fractions, and those labelled in blue showed significant association with F6. Labels in black show no significant association. Significance was determined by Monte Carlo Chi-squared test (p<0.001).

As vesicles are membrane bound structures, fractions enriched for these should also be enriched for membrane proteins. To determine if this were the case, the proteins present in the fractions were classed as either membrane or soluble through DeepLoc prediction^28^. Soluble proteins were further subdivided into those with a signal peptide (+ SP) and those without (-SP) (Fig. 7a). Signal peptide containing proteins are targeted to the ER where most are secreted via the conventional ER-to-Golgi pathway, although exceptions whereby the Golgi is by-passed (unconventional secretion) have also been observed (reviewed in Nickel et al.^29^ and Grieve et al.^30^). In the background set of proteins, almost a quarter were predicted to be membrane localised (24.3%, 1,626 proteins), with the remaining three quarters consisting of soluble proteins. Compared to this, the buoyant fractions were enriched in membrane proteins (62.2%, 952 proteins), whilst F6 was clearly depleted (1.3%, 8 proteins). Although F6 consisted mainly of soluble proteins, representing 98.7% of all proteins present in this fraction compared to 37.7% in the buoyant fractions, only 1.9% of these (12 proteins) contained a signal peptide, compared to 10.4% (159) in the buoyant fractions (Supplementary Data 2). The background set of proteins consisted of 6.1% (409) soluble proteins with signal peptide.

### Effector proteins are associated with buoyant fractions

Since the RXLR effector PITG_04314 and the apoplastic effector/pectin esterase PITG_01029 were associated with the vesicle-rich buoyant fractions, we set out to determine if this was a general trend for effector proteins in our dataset. Of the annotated 596 RXLR effectors in the *P. infestans* database, 58 were detected in our dataset, 31 (53.4%) of which were enriched within the buoyant fractions, and only one (1.7%) enriched within F6 (Fig. 7b). For carbohydrate active enzymes (CAZymes), which include pectinesterases, 505 were predicted to be encoded in the *P. infestans* genome; 181 of these were detected in our dataset, of which 90 (49.7%) were enriched in the buoyant fractions, and one (0.5%) enriched within F6 (Fig. 7b).

These findings together suggest buoyant fractions enrich for membrane proteins and for signal peptide containing soluble proteins which enter the secretory pathway, such as RXLR effectors and CAZymes, whereas F6 is enriched for non-secretory soluble proteins.

To determine whether our data set captured *bona fide* extracellular secreted proteins, we compared our data set to a published *P. infestans *extracellular secretome. Meijer et al.^31^ previously profiled the extracellular proteome of *P. infestans* mycelia grown in seven different types of media and collectively identified 283 extracellular *P. Infestans* proteins. The majority of these proteins were those involved in defence against oxidative stress, pathogen associated proteins such as effectors and elicitins, and various proteases (aspartic, cysteine, and metalloproteases). Of these 283 proteins, over half (164 proteins) were detected in our proteomics data set, of which 47.5% (78 proteins) were enriched within the buoyant fractions, with only 1.2% (two proteins) enriched within F6 (Fig. 7b, Supplementary Data 2).

### Buoyant gradient ultracentrifugation separates proteins associated with different cellular compartments

To obtain an overview of the intracellular sources of the proteins found within the fractions, the enriched proteins were sub-divided into their predicted cellular localisations and compared.

This analysis revealed significant association between the type of fraction (buoyant vs. dense) and the predicted cellular localisations of the proteins present, except for proteins associated with peroxisomes which was similar between both types of fractions (Fig. 7c). Proteins associated with the nucleus (43.5%) and cytoplasm (40.9%) represented the most abundant types of proteins in F6. Proteins associated with cell membrane, Golgi apparatus, and the extracellular compartment collectively formed less than 1% of the enriched proteins in F6, and no proteins associated with lysosome/vacuole or endoplasmic reticulum were identified as enriched in this fraction. In complete contrast, the buoyant fractions were depleted in cytoplasmic (12.4%) and nuclear proteins (6.1%) but were enriched for those associated with cell membrane (25.1%), endoplasmic reticulum (15%), extracellular (8.3%), Golgi apparatus (4.4%) and lysosome/vacuole (4.3%), totalling 57.2% of the total enriched proteins.

The buoyant fractions were also more enriched in mitochondrial proteins compared to F6 (20.7% vs. 11.4%, respectively). However, the nature of the mitochondrial proteins enriched differed – 66.1% of mitochondrial proteins enriched in the buoyant fraction were predicted to be membrane-associated, whereas only 7% of mitochondrial proteins enriched in F6 had this property, with the majority predicted to be soluble (Supplementary Fig. S7).

### Gene ontology analysis reveals enrichment for proteins associated with membranes and vesicle trafficking in buoyant fractions

Since the data suggested the buoyant fractions and the denser F6 fraction differ in the general types of proteins enriched and their subcellular origins, we used Gene Ontology (GO) analysis to determine whether these two sets of proteins also differed in their associated biological functions.

Both the 1,528 proteins enriched in the buoyant fractions and the 625 proteins enriched in F6 were compared against the background set of all 6,685 detected proteins to look for enrichment of GO terms belonging to the Biological Process category.

**Fig. 8:**
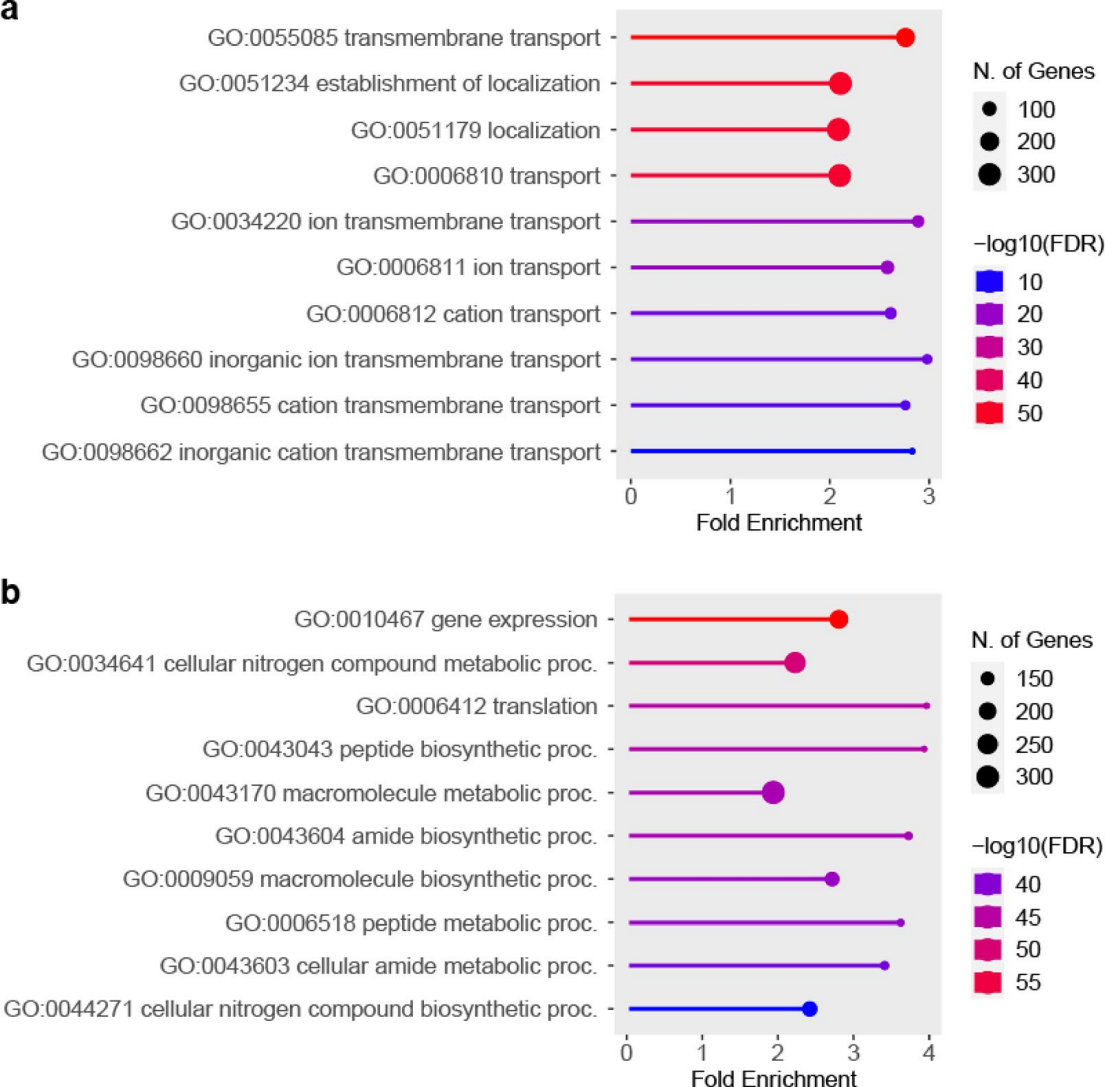
Top ten Gene Ontology terms enriched in the buoyant fractions and F6 as assessed by ShinyGO. (**a**) Biological Process GO terms enriched in the buoyant fractions F2, F3 and F4. (**b**) Biological Process GO terms enriched in fraction F6. Images were generated in ShinyGO v0.81^51^.

‘Transmembrane transport’ (GO:0055085, 203 proteins), ‘Establishment of localisation’ (GO:0051234, 322 proteins), and ‘Localisation’ (GO:0051179, 327 proteins) were identified as the top three GO terms for the buoyant fractions (Fig. 8a, Supplementary Data 3). For F6, ‘Gene expression’ (GO:0010467, 221 proteins), ‘Cellular nitrogen compound metabolic process’ (GO:0034641, 265 proteins), and ‘Translation’ (GO:0006412, 268 proteins) were identified as the top three GO terms (Fig. 8b, Supplementary Data 4).

Since proteins can be assigned more than one GO term, with large redundancy between some terms, the top three GO terms were collapsed to give a single non-redundant protein list for further analysis. This resulted in a collapsed set of 327 proteins for the buoyant fractions (Supplementary Data 3), and 268 proteins for F6 (Supplementary Data 4).

Interrogation of the proteins enriched in the buoyant fraction included a range of membrane trafficking proteins, such as 16 Ras-associated binding (Rab) proteins, eight vacuolar protein sorting associated (Vps) proteins, 17 soluble *N*-ethylmaleimide-sensitive factor attachment protein receptor (SNARE) proteins and proteins related to SNARE function, six Golgi Dynamics (GOLD) domain-containing proteins, and various other proteins involved in vesicle trafficking (Supplementary Data 3).

**Fig. 9:**
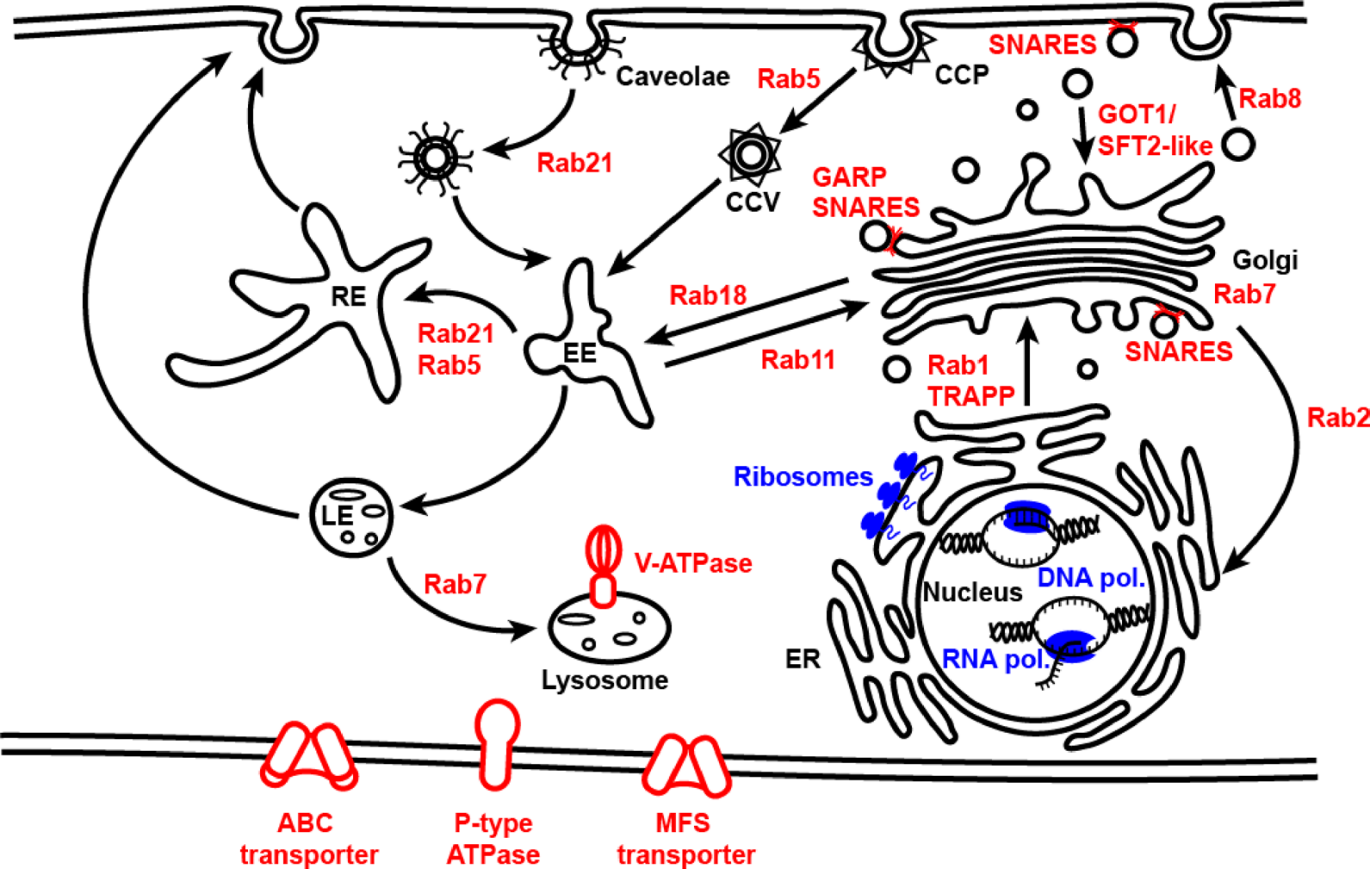
Overview of cellular localisation of enriched proteins from GO analysis. Proteins enriched in buoyant fractions (red) and in F6 (blue) are associated with distinct cellular compartments. CCP: clathrin-coated pit, CCV: clathrin-coated vesicle, RE: recycling endosome, EE: early endosome, LE: late endosome, ER: endoplasmic reticulum. Image drawn in Adobe Illustrator.

Membrane transporters/proton pumps were also enriched in the buoyant fractions, with proteins forming V-ATPases (six proteins), ABC transporters (41 proteins), MSF transporters (33 proteins), and P-type ATPases (18 proteins) identified. Figure 9 summarises the cellular localisations of the enriched proteins.

In contrast, the proteins enriched in the dense F6 fraction were dominated by ribosomal subunits and ribosome-associated proteins, DNA and RNA polymerases/proteins involved in transcription, translation, DNA repair and replication, and ribonuclear proteins (Fig. 9, Supplementary Data 4).

### Buoyant density ultracentrifugation separates intracellular vesicles with divergent protein content

To determine whether the present protocol could separate proteins based on their association with intracellular vesicles of differing buoyancy characteristics, we compared protein content of the buoyant fractions F2 (1.111 ± 0.013 g/mL) and F4 (1.150 ± 0.012 g/mL). We chose these two fractions for comparison as they represent the extreme ends of the buoyant fractions analysed here in terms of density.

**Fig. 10:**
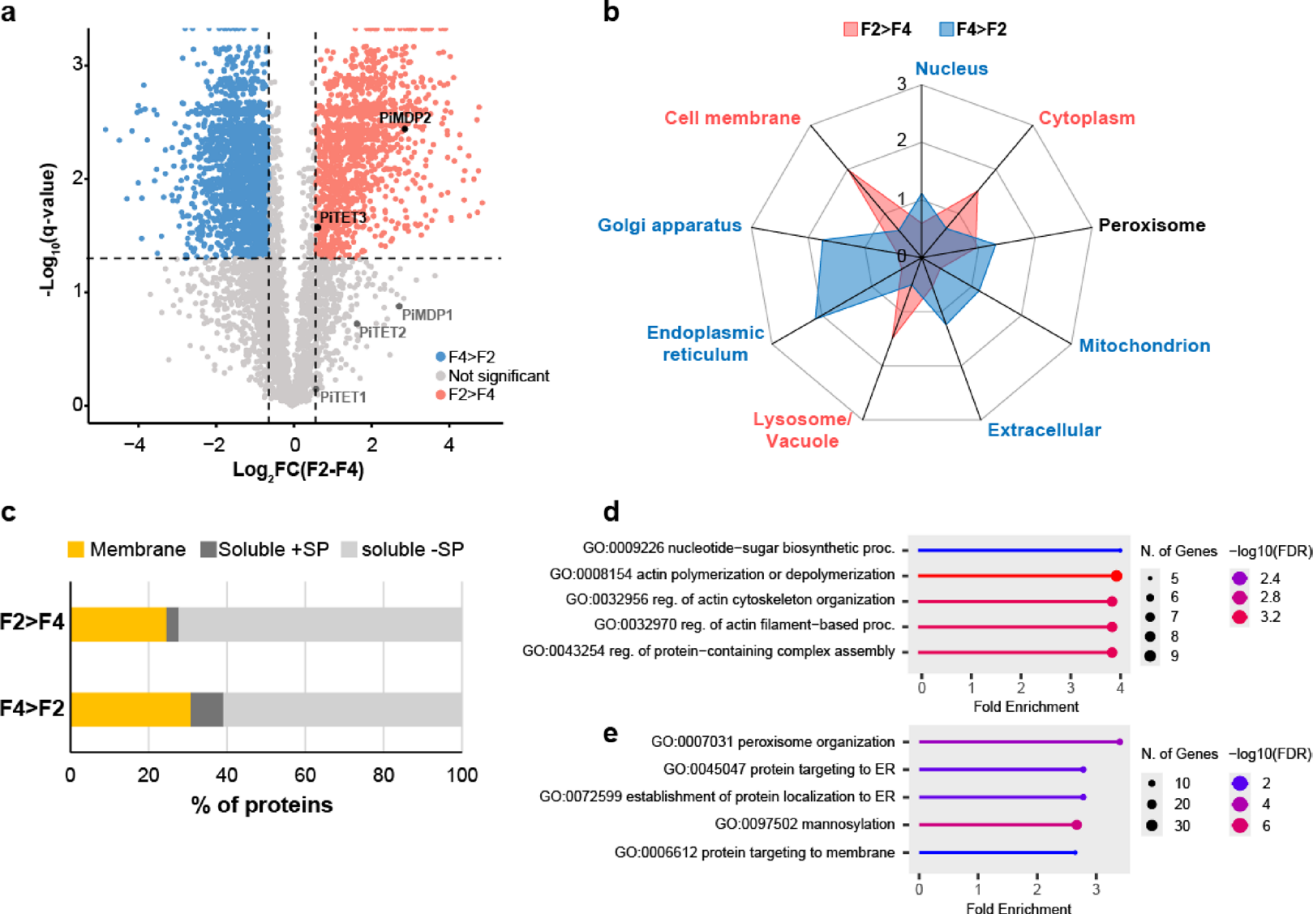
Comparison of proteins present in buoyant fractions F2 and F4. (**a**) Volcano plot showing proteins differentially abundant between F2 and F4, defined as those with −0.6 ≤ Log_2_FC(F2-F4) ≥ 0.6 and q-value <0.05. The positions of three *P. infestans* tetraspanins (PiTET1 (PITG_03954), PiTET2 (PITG_03950) and PiTET3 (PITG_12224)), and two MARVEL proteins (PiMDP1 (PITG_13660) and PiMDP2 (PITG_13661)) are highlighted in the plot. (**b**) Radar chart showing percentage of proteins associated with each cellular compartment (predicted by DeepLoc) as a ratio of the percentage found in the background set (fold-enrichment) for proteins enriched in F2 (F2> F4, pink filled square) and proteins enriched in F4 (F4> F2, blue filled square). Cellular localisations labelled in pink showed significant association with fraction F2, and those labelled in blue showed significant association with F4. Labels in black show no significant association. Significance was determined by Monte Carlo Chi-squared test (*p*<0.001). (**c**) Percentage of proteins predicted membrane associated or soluble found enriched in F2 and F4. The predicted soluble proteins were further subdivided into those with (‘soluble +SP’) and without (‘soluble -SP) a signal peptide. (**d**) Top five GO terms for Biological Process enriched in fraction F2 over F4 (image generated in ShinyGO v0.81^51^). (**e**) Top five GO terms for Biological Process enriched in fraction F4 over F2 (image generated in ShinyGO v0.81^51^).

Analysis of differential protein abundance identified 1,330 and 1,909 proteins which were significantly more abundant in F2 over F4 (F2 > F4) and in F4 over F2 (F4 > F2), respectively (Fig. 10a). Amongst the differentially abundant proteins was the tetraspanin-like PiTET3 (PITG_12224), and the MARVEL domain protein PiMDP2 (PITG_13661), which were significantly more abundant in F2. In addition, 44 and 75 proteins were found to be exclusive to F2 and F4, respectively. This gave a combined total of 1,374 and 1,984 proteins with a higher association with F2 and F4, respectively (Supplementary Data 5).

DeepLoc prediction of cellular localisations indicated proteins enriched in F2 had a higher association with the cell membrane, lysosomes and cytoplasm, whereas proteins more abundant in F4 were associated with the endoplasmic reticulum, Golgi, extracellular compartment, mitochondria and nucleus (Fig. 10b). F4 was also found to contain more membrane and signal peptide containing proteins than F2; 610 proteins (30.7%) versus 337 proteins (24.5%), and 165 proteins (8.3%) versus 44 proteins (3.2%), respectively (Fig. 10c).

GO analysis of the proteins in fractions F2 and F4 also showed differences in the Biological Process GO terms enriched. The top three GO terms for F2 were ‘nucleotide-sugar biosynthetic process’ (GO:0009226), ‘actin polymerization or depolymerization’ (GO: 0008154), and ‘regulation of actin cytoskeleton organization’ (GO:0032956) (Fig. 10d). For F4, the top three GO terms were ‘peroxisome organization’ (GO:0007031), ‘protein targeting to ER’ (GO:0045047), and ‘establishment of protein localization to ER’ (GO:0072599) (Fig. 10e).

## Discussion

In this study, we developed a method for isolating intracellular vesicles from the late blight pathogen *P. infestans* and identified associated proteins by DIA-mass spectrometry. We employed cushioned bottom-loaded gradient ultracentrifugation to successfully isolate intracellular vesicles, based on the observations that (i) proteins migrating to the buoyant fractions F2, F3 and F4 coincided with the presence of vesicles; (ii) the virtual absence of vesicles in the dense fraction F6; and (iii) the absence of both protein migration and vesicle presence on pretreatment of the buoyant fractions with the detergent Triton X-100, coinciding with increased protein accumulation in the denser fractions, including F6. Together, these observations suggest that intracellular vesicles migrate up the density gradient to fractions of lower density, separated from free proteins/protein aggregates and cellular debris, which accumulate at the bottom of the gradient in higher density fractions.

Mass spectrometry revealed homologues of well-characterised membrane and vesicle marker proteins (e.g., TETs, Rabs, SNAREs) commonly associated with EVs were enriched within the buoyant fractions. This suggests capture of vesicles during their biogenesis and trafficking within the cell. In addition to the membrane-associated proteins, the secreted effectors PITG_04314 and PITG_01029 were also identified in the buoyant fractions, indicating soluble cargo proteins were also isolated using this method. The robust dataset presented here provides a valuable resource that can be mined for potential membrane and cargo markers from *P. infestans* intracellular vesicles. Additionally, our method can be adapted for future studies to characterise vesicles with specific buoyant properties in detail.

In this study, we focused analysis on proteins commonly enriched in three buoyant fractions over the denser fraction F6. Rab proteins are well known for their role in intracellular vesicle trafficking^32,33^, with their presence here suggesting enrichment of diverse intracellular vesicles: Rabs associated with ER-Golgi transport (Rab 1, 2, 18), trans-Golgi network (TGN)-plasma membrane transport (Rab 6, 8, 11), early endosomes mediating clathrin (Rab5) and caveolin (Rab21) pathways^34^, and late endosomes/lysosome/phagosome (Rab7, Rab 32) were all identified in the buoyant fractions.

Rabs can act as markers for recognition of target membranes during vesicle docking and membrane tethering prior to the initiation of fusion. This recognition is mediated by tethering factors, such as the Golgi-localised complex Vps52/53/54 (homologues of all three of which were identified here) which recognises Ypt6p, the yeast homologue of Rab6. This recognition is required for the initial tethering of vesicles to the trans-Golgi network during retrograde trafficking, bringing the membranes into close proximity for subsequent fusion mediated by SNAREs^35–37^.

SNAREs mediate the majority of membrane fusion events during exocytosis and endocytosis; they are characterised by 60-70aa SNARE motifs, of which there are four types, Qa, Qb, Qc and R, distributed between vesicle and target membranes, bringing the opposing membranes together on interaction to form the SNARE complex^37^.

Interestingly, the protein content of the three buoyant fractions were not completely overlapping; this suggests the method used here can separate distinct vesicle populations, as also indicated by the presence of numerous Rabs associated with diverse intracellular vesicle populations. In support of this, comparison between buoyant fractions F2 and F4, representing the extremes of the buoyant fractions analysed in this study, showed considerable differences in the proteins associated with them. Of particular interest are the potential vesicle membrane markers identified amongst these proteins. Present in both fractions were three of the five *P. infestans* tetraspanin homologs; PiTET1 (PITG_03954), PiTET2 (PITG_03950) and PiTET3 (PITG_12224), with PiTET3 significantly more abundant in fractions F2. Similarly, two transmembrane MARVEL domain proteins, PiMDP1 and PiMDP2 (PITG_13660 and PITG_13661, respectively) identified in *P. infestans* EVs^16^ were also found in fractions F2 and F4, with PiMDP2 enriched in fraction F2. Unfortunately, the tetraspanin homologs and MARVEL domain proteins were not confirmed as EV markers before the completion of this study and so were not utilised here. Although we cannot exclude the possibility of contamination of our intracellular preparation with EVs, the detection of known EV markers was not unexpected – we anticipated the capture of vesicles destined for extracellular secretion during biogenesis and enroute to secretion. Breen et al.^16^ were able to visualise fluorescently tagged MARVEL proteins PiMDP1 and PiMDP2 localising to numerous punctate structures in *P. infestans*, with PiMDP2 co-localising with PITG_04314 during *in vitro* growth and during infection, and both proteins accumulating at haustoria, the site of PITG_04314 secretion. This suggests that the MARVEL domain proteins detected in this study derive from isolated intracellular vesicles, demonstrating that tracking of soluble cargo protein, in the absence of an available vesicle marker, is a valid strategy for the isolation of secretion-competent vesicles.

To further separate sub-populations of vesicles, collection and analysis of more refined fractions over a larger portion of the gradient to reveal the densities at which PiTET1, PiTET2 and PiMDP1 accumulate would be required. From our data, there was an observed tendency for accumulation of PITG_01029-mCitrine to peak at a slightly higher density than PITG_04314, hinting at a possible diversification of vesicles (Figs. 3 and 4) and, indeed, these effector proteins tend not to colocalise at the same punctate structures in hyphae^16^. The present data demonstrates how this method could be applied to separate distinct vesicle populations associated with specific membrane markers and cargoes. This would aid in the better understanding of the process of protein secretion in *P. infestans* and the development of novel control methods aimed at selectively disrupting this process.

Looking forward, epitope tagging of potential vesicle membrane markers identified in this study would enable further enrichment and purification. Immunoprecipitation of the gradient fractions in which they are most abundant would enable interrogation of protein cargo. Pairing this with markers for specific intracellular localisations, such as the Rab proteins, presents the possibility of identifying the intracellular pathways through which proteins are packaged, trafficked and secreted.

Combining this work with *Phytophthora infestans* EV proteomics will be a powerful tool to further unravel how effector proteins are sorted, transported and secreted. This would enable the exciting possibility of tracking effectors from biogenesis, to secretion, and to delivery to their final destinations *in planta*. Improving our understanding of how *Phytophthora infestans* infects the potato host will reveal new targets against which pathogen management strategies can be developed and tested to curb the devastating crop losses caused by this economically important plant pathogen.

## Methods

### *Phytophthora infestans* in vitro culture

For culturing *in vitro* on solid media, *P. infestans* strain 3928A was cultured on rye agar plates supplemented with 100 µg/mL ampicillin and 10 µg/mL pimaricin at 19 °C in the dark for 11 to 14 days for sporulation^38^. For transformants, plates were additionally supplemented with 10 µg/mL G418.

For culturing in liquid media, sporangia from *P. infestans* were collected by flooding plates with amended Lima bean broth (ALB)^38^ supplemented with 100 µg/mL ampicillin (and additionally 2.5 µg/mL G418 for transformants) and scraping the plates with a spreader. The media was then filtered through a 70 µM nylon mesh to remove mycelial fragments.

For confirmation of expression and secretion of fluorescently tagged proteins in transformants, sporangia were collected from two 9 cm diameter Petri dish cultured into a total of 10 mL ALB as described above. Filtered sporangia were incubated in the dark for three days at 19 °C. Mycelia were then transferred to 1 mL clarified ALB (centrifuged at 10,000 × g for 2 h to remove particulate matter) and incubated in the dark for a further 24 h, after which the mycelia and condition media (CM) were collected. Mycelia were dried on tissue to remove excess media, and proteins in the CM were chloroform-methanol precipitated. 2 × SDS loading buffer was added directly to the mycelial sample (60 µl) and to the chloroform-precipitated protein pellet (40 µl) and processed for western blotting as described below.

For culturing for intracellular vesicle isolation, sporangia from 42 × 15 cm diameter Petri dish cultures were collected and used to inoculate 6 × 100 mL of clarified ALB as described above. Cultures were grown at 19 °C in the dark for five days and mycelia collected and processed for intracellular vesicle isolation as detailed below.

### Expression of fluorescently tagged effectors in *P. infestans*

A dual expression vector for simultaneous expression of both an mCherry tagged protein with an mCitrine tagged protein was generated by modification of the vector pmCherryN^39^. mCitrine was amplified by PCR from vector pmCitrine_N1 (Addgene, plasmid #92420) to introduce the restriction endonuclease sites ClaI-SpeI-AsiSI flanking the N- and C-termini of mCitrine, respectively. The PCR product was cloned into the vector pTOR (NCBI GenBank accession EU257520) between the ClaI and EcoRI restriction sites. The pTOR vector contains a Ham34 promoter (HamP) and a Ham34 terminator (HamT) up- and downstream of the multiple cloning site (respectively) for expression in *Phytophthora.* The insert with the upstream HamP and downstream HamT was cloned with primers designed to introduce flanking SacI sites; this SacI-HamP-ClaI-SpeI-AsiSI-mCitrine-XbaI-FseI-EcoRI-HamT-SacI expression cassette was inserted into the SacI site of pmCherryN to generate the dual expression vector pDual_mCherryN_mCitrine (Supplementary Fig. S1).

PITG_04314 was cloned between the AgeI/PacI restriction sites upstream of the mCherry gene, and PITG_01029 was cloned between the ClaI/AsiSI restriction sites upstream of mCitrine. All primers used are listed in Supplementary Table 1.

The dual vector containing PITG_04314 and PITG_01029 was transformed into *P. infestans* protoplasts using a polyethylene glycol (PEG)-CaCl_2_-lipofectin mediated method^38,40^.

### Plant growth conditions

*Nicotiana benthamiana* was grown in commercially available compost under glasshouse conditions of 16/8 h light/dark cycle with a maximum daytime temperature of 26 °C and night-time temperature of 22 °C. Plants were used at 4–5 weeks old.

### *P. infestans* inoculation of *N. benthamiana*

*P. infestans* sporangia were harvested in sterile distilled water from 11 to 14 days old cultures, passed through a 70 μm cell sieve (Corning), and the concentration adjusted to 5 × 10^5^ sporangia per mL for use as inoculum. The underside of detached *N. benthamiana* leaves were nicked with a scalpel blade and a 10 µL drop of inoculum deposited at this site. The leaves were incubated at 19 °C in a humid box and imaged by confocal microscopy at five days post inoculation.

### Confocal microscopy

*N. benthamiana* leaves infected with transgenic *P. infestans* co-expressing PITG_04314-mCherry and PITG_01029-mCitrine were mounted on slides and imaged using a Nikon A1R confocal microscope with a 40 × water immersion lens. mCherry was imaged using 561 nm excitation and emissions collected between 570 and 620 nm. mCitrine was imaged with 488 nm excitation and emissions collected between 500 and 530 nm.

### Intracellular vesicle isolation

Mycelia grown in 100 mL liquid culture (as described above) was collected using a filter unit fitted with 70 μm nylon mesh and rinsed twice with cold isolation buffer adapted from Heard et al.^41^ (150 mM Na-HEPES, 10 mM M EDTA, 10 mM EGTA, 17.5% (w/v) sucrose, 7.5 mM KCl, 1 × protease inhibitor (Roche)). Washed mycelia were ground with a pestle and mortar with 11 mL isolation buffer and filtered through a 40 μm cell sieve to remove large debris. The extract was pre-cleared by differential centrifugation at 2,000 × g for 10 min, followed by centrifugation at 10,000 × g for 30 min before cushioned ultracentrifugation of the supernatant. All steps were performed on ice or at 4 °C.

### Cushioned bottom-loaded density gradient ultracentrifugation

The following method was developed based on the methods of Duong et al.^19^, Crescitelli et al.^4^, and Rutter et al.^25^. 10 mL of pre-cleared extract was overlaid onto a 2 mL 60% Iodixanol/OptiPrep (STEMCELL Technologies) cushion in a 14 × 89 mm polycarbonate open top tube (Beckman Coulter) and centrifuged at 100,000 × g for 2 h in a Beckman Coulter Optima L-80 XP ultracentrifuge with a swinging bucket rotor (SW41 Ti). After centrifugation, all but 1 mL of extract above the cushion was discarded. The remaining 1 mL of extract was combined with the 2 mL iodixanol cushion and transferred to the bottom of a new ultracentrifuge tube. The sample was overlaid sequentially with 1.3 mL each of 35%, 30%, 25%, 20%, 17.5%, 15% and 10% iodixanol to form a discontinuous gradient and centrifuged at 120,000 × g for 20 h without braking during deceleration. Starting from the top of the tube, eight 1.5 mL fractions (F1-F8) were collected from the gradient for further analysis and processing. All steps were performed on ice or at 4°C. OD 340 nm readings from fractions were measured using a Nanodrop1 spectrophotometer and densities calculated using a standard curve of iodixanol dilutions of known densities.

### Triton X-100 treatment

1% Triton X-100 was added to the 1 mL extract combined with the 2 mL iodixanol cushion after cushioned ultracentrifugation (described above) and incubated on ice for 30 min before overlaying with iodixanol as above for gradient ultracentrifugation. A control sample which was incubated on ice for 30 min without the addition of 1% Triton X-100 was run in parallel.

### Deglycosylation assay

Mycelia and culture medium of the dual transformant grown in 75 mL of clarified ALB for five days was collected. Mycelia were ground in 5 mL of TEN buffer (25 mM Tris-HCl, pH 7.5, 1 mM EDTA, 150 mM NaCl) and centrifuged for 10 min at 3,500 × g to remove cellular debris. 5 ml mycelial extract and 5 ml of the culture medium were precipitated by chloroform-methanol extraction and the protein pellet resuspended in 500 µl sterile distilled water. 40 µl of each sample was treated with Protein Deglycosylation Mix II (NEB) under denaturing conditions following the manufacturer’s instructions. 2 × LDS loading buffer was added to each sample and analysed by western blot as described below.

### Western blotting

Protein samples in 2 × SDS loading buffer were incubated at 95 °C for 10 min; protein samples in 2 × LDS loading buffer were incubated at 70 °C for 10 min. Samples were separated on 12% acrylamide tris-glycine gels and transferred onto 0.45 μm nitrocellulose membranes (Amersham). Total proteins on membranes were visualised using Revert 700 Total Protein Stain Kit (LI-COR) following manufacturer’s instructions. Membranes were blocked in 4% milk in PBS prior to probing. For histone, membranes were probed with rabbit anti-Histone H3 (abcam) and subsequently with IRDye^®^ 800CW Goat anti-Rabbit IgG secondary antibody (LI-COR). For simultaneous detection of PITG_04314-mCherry and PITG_01029-mCitrine, membranes were probed with both rat anti-mRFP (Chromotek) and mouse anti-GFP (Roche), respectively. Membranes were then simultaneously probed with secondary antibodies IRDye^®^ 800CW Goat anti-Rat IgG and IRDye^®^ 680CW Goat anti-Mouse IgG (LI-COR). Fluorescence signal from the IRDye^®^ 680CW and IRDye^®^ 800CW conjugated secondary antibodies were imaged in the 700 nm and 800 nm channels (respectively), either separately (for histone) or simultaneously (for PITG_04314-mCherry and PITG_01029-mCitrine), with the LI-COR Odyssey CLx Near-Infrared Fluorescence Imaging System. Images were analysed/quantified using Image Studio Lite version 5.2.5 (LI-COR).

### Transmission electron microscopy of intracellular vesicles in gradient fractions

Samples were fixed with 2% formaldehyde and 1% glutaraldehyde onto carbon coated formvar on 300 mesh nickel grids (Agar Scientific). Samples were then stained with 4% uranyl oxylate (pH 7), embedded with 0.15% methyl cellulose and 0.4% uranyl acetate, then dried and stored at room temperature. Grids were imaged with a Jeol JEM 1400 transmission electron microscope.

### Processing of gradient fractions for mass spectrometry

Equivalent fractions from six gradients performed simultaneously for fractions F2, F3, F4 and F6 were pooled to form a single replicate experiment, with each replicate experiment performed on separate occasions. Proteins were chloroform-methanol precipitated, and protein pellets resuspended in 200 µl 1% sodium deoxycholate (Sigma-Aldrich), 50 mM Tris-HCl, pH 8. Proteins were reduced with 5 mM DTT, alkylated with 18.75 mM Iodoacetamide, and digested with 2 µg Trypsin/Lys-C mix (Promega) overnight at 37 °C in the presence of 1% sodium deoxycholate. The samples were then further digested with 1 µg Trypsin Platinum (Promega) for 3 h. Detergent was removed from the peptides by ethyl acetate extraction^42^ followed by processing with HiPPR™ Detergent Removal Spin Column Kit (Thermo Fisher Scientific). Mass spectrometry of the samples was performed by the FingerPrints Proteomics Facility at the University of Dundee.

### DIA LC-MS/MS

Analysis of peptide readout was performed on a Q Exactive HF, Mass Spectrometer (Thermo Scientific) coupled to a Dionex Ultimate 3000 RS (Thermo Scientific). LC buffers used are the following: buffer A (0.1% formic acid in Milli-Q water (v/v), buffer B (80% acetonitrile and 0.1% formic acid in Milli-Q water (v/v), loading buffer (0.1% TF in Milli-Q). Aliquots of 5 or 10 µL desalted peptides were loaded at 10 µL/min onto a trap column (100 μm × 2 cm, PepMap nanoViper C18 column, 5 μm, 100 Å, Thermo Scientific) equilibrated in 0.1% TFA for 15 min. The trapping column was washed for 6 min at the same flow rate with 0.1% TFA and then switched in-line with µPAC Neo, 110 cm, nanoLC column, equilibrated at a flow rate of 300nl/min for 30 min. The peptides were eluted from the column at a constant flow rate of 300 nl/min with a linear gradient from 1% buffer B to 7% buffer B in 6 min, from 7% B to 26% buffer B in 117 min, from 26% buffer B to 36% buffer B within 23 min and then from 46% buffer B to 98% buffer B in 10 min. The column was then washed with 98% buffer B for 18 min. Two blanks were run between each sample to reduce carry-over. The column was kept at a constant temperature of 50 °C.

Q-exactive HF was operated in positive ionization mode using an easy spray source. The source voltage was set to 2.2 Kv and the capillary temperature was 250 °C. Data were acquired in Data Independent Acquisition Mode as previously described^43^, with little modification. A scan cycle comprised a full MS scan (*m/z* range from 345 − 155), resolution was set to 60,000, AGC target 3 × 10^6^, maximum injection time 200 ms. MS survey scans were followed by DIA scans of dynamic window widths with an overlap of 0.5 Th. DIA spectra were recorded at a resolution of 30.000 at 200 *m/z* using an automatic gain control target of 3 × 10^6^, a maximum injection time of 55 ms and a first fixed mass of 200 *m/z*. Normalised collision energy was set to 25% with a default charge state set at 3. Data for both MS scan and MS/MS DIA scan events were acquired in profile mode.

### Data analysis

The Thermo raw files were analysed using Spectronaut (version 17.4.230317.55965, Biognosys, AG). The directDIA workflow, using the default settings (BGS Factory Settings) with the following modifications: decoy generation set to inverse; Protein LFQ Method was set to QUANT 2.0 (SN Standard) and Precursor Filtering set to Identified (Qvalue); Cross-Run Normalization was unchecked; Precursor Qvalue Cutoff and Protein Qvalue Cutoff (Experimental) set to 0.01; Precursor PEP Cutoff set to 0.01, Protein Qvalue Cutoff (Run) set to 0.01 and Protein PEP Cutoff set to 0.01.

For the Pulsar search the settings were: maximum of two missed trypsin cleavages; PSM, Protein and Peptide FDR levels set to 0.01; scanning range set to 300-1,800 m/z and Relative Intensity (Minimum) set to 5%; cysteine carbamidomethylation set as fixed modification and acetyl (N-term), deamidation (asparagine, glutamine), dioxidation (methionine, tryptophan), glutamine to pyro-Glu, oxidation of methionine and Phospho (STY) set as variable modifications. The databases used were *P. infestans* T30-4, downloaded from fungidb.org on 06–12-2023 (17,799 entries) modified with the sequences for mCherry and mCitrine, and *P. vulgaris* proteome, downloaded from uniprot.org (30,501 entries, UP000000226).

The resulting protein groups table was analysed in Perseus v2.0.11^44^. From here on, the term “protein” is used interchangeably with “protein group” for simplicity. Proteins matching *Phaseolus vulgaris* proteins were removed as potential contamination from ALB media. Raw intensity values of the remaining protein groups for each sample were Log_2_ transformed and normalised to the median intensity within the sample prior to statistical analysis. At this point, one replicate experiment was excluded as an outlier, with the remaining three replicates taken forward for further statistical analysis as below.

Proteins classed as detected in the entire dataset were those present in at least two of all samples. Proteins classed as detected within an individual fraction were those detected in at least two biological replicates of that fraction. The more stringent classification of present or absent within a fraction required a protein group to be present or absent in all three biological replicates of that fraction, respectively.

For comparison of protein abundance between fractions F2, F3, F4 and F6, proteins detected (as defined above) in all four fractions were used for the analysis. One-way ANOVA and Tukey’s HSD test (FDR < 0.05 and q-value < 0.05) were used to identify proteins significantly differentially abundant between fractions. Proteins were then further filtered on −0.6 ≥ Log_2_ fold-change (FC) ≥ 0.6, equivalent to a fold-change of at least 1.5 in the negative or positive direction, respectively, or for presence/absence. These filtered proteins were combined to form a set of high confidence proteins taken forward for further analysis.

For comparison of fraction F2 against F4, proteins detected (as defined above) in either F2 or F4 were used for the analysis. Two-sample t-test (FDR < 0.05 and q-value < 0.05) was used to identify proteins significantly differentially abundant between the fractions. Proteins were then further filtered on −0.6 ≥ Log_2_(FC) ≥ 0.6, equivalent to a fold-change of at least 1.5 in the negative or positive direction, respectively.

Euler’s diagrams, heatmaps, and volcano and violin plots were drawn using the R packages ‘eulerr’^45^ (version 7.0.2), ‘gplots’^46^ (version 3.2.0), and ‘ggplot2’^47^ (version 3.4.3), respectively.

### *In silico* prediction of protein cellular localisation and carbohydrate active enzymes

The predicted *P. infestans* T30-4 proteome was analysed using the DeepLoc 1.0 server (https://services.healthtech.dtu.dk/service.php?DeepLoc-1.0)^28^ and the dbCAN3 server (https://bcb.unl.edu/dbCAN2/)^48^ to predict protein cellular localisation and to identify potential carbohydrate active enzymes (CAZymes), respectively.

Four proteins were omitted from the DeepLoc anlaysis due to excessive length (PITG_01932, PITG_04003, PITG_04980 and PITG_12547). DeepLoc predictions for Plastids were combined with Mitochondrion, as *P. infestans* does not contain plastids. Counts for cellular localisation were performed on the individual protein level (e.g., proteins within protein groups were counted individually) and statistical associations were assessed by Pearson’s Chi-squared test with Monte Carlo simulation (100,000 simulations) using the R package “chisq.posthoc.test” with Bonferroni-Holm correction for multiple testing^49^.

### Identification of signal peptide containing proteins and RXLRs

To identify the presence of signal peptide containing proteins, protein lists were searched against a reference list of 2,365 predicted signal peptide-containing proteins within the *P. infestans* genome. This reference list was generated by combining predicted signal peptide containing protein lists from the FungiDB website (https://fungidb.org/fungidb/app/, release 68), Meijer et al.^31^, and Raffaele et al.^50^ (Supplementary Data 2). The presence of RxLR effector proteins was determined by sub-setting proteins with the term ‘RxLR’ within the ‘ProteinDescription’ field.

### Gene ontology analysis

Gene Ontology (GO) analysis was performed on the individual protein level in ShinyGO v0.81^51^ available at https://bioinformatics.sdstate.edu/go using the set of 6,685 all detected proteins as the background set, with species database set to *Phytophthora infestans* genes ASM14294v1. The analysis was set to return the top 10 pathways for Biological Process, with a minimum pathway size of 10. All other settings were left at default, with FDR set to 0.05. For lollipop diagrams of enriched GO terms, top GO terms with a minimum of five proteins identified were displayed. In some cases where redundancy between GO terms was high, proteins in the top three GO terms were collapsed into a single non-redundant list. Interpro and Pfam protein domain descriptions obtained from FungiDB (https://fungidb.org/fungidb/app/, release 68) were used to infer protein descriptions/function where annotation was lacking.

## Supplementary Information

Below is the link to the electronic supplementary material.

Supplementary Material 1

Supplementary Material 2

Supplementary Material 3

Supplementary Material 4

Supplementary Material 5

Supplementary Material 6

## Data Availability

Data analysed to produce the results presented are included in the supplementary data. The mass spectrometry proteomics data have been deposited to the ProteomeXchange Consortium via the PRIDE^52^ partner repository with the dataset identifier PXD072961.
