# Supplementary material for: Isolation and proteomic analysis of intracellular vesicles from the potato late blight pathogen Phytophthora infestans
Source: Sci Rep. 2026 Jan 25;16:6185. doi: 10.1038/s41598-026-37161-2 (PMC12905231; doi:10.1038/s41598-026-37161-2)
Supplement: Supplementary file 6 — Supplementary Material 6 [file 41598_2026_37161_MOESM6_ESM.docx]

# Isolation and proteomic analysis of intracellular vesicles from the potato late blight pathogen *Phytophthora infestans*

Jasmine Pham^1,2,*^, Stephen C. Whisson^3^, Charlotte H. Hurst^2,4^, Sean Chapman^2^, Paul R. J. Birch^2,3,5,*^

^1^Division of Molecular Cell and Developmental Biology, School of Life Sciences, University of Dundee, DD2 5DA, UK

^2^Division of Plant Sciences, University of Dundee, at The James Hutton Institute, Errol Rd, Invergowrie, Dundee, DD2 5DA, UK

^3^Department of Cell and Molecular Sciences, The James Hutton Institute, Invergowrie, Dundee, DD2 5DA

^4^Medical Research Council Protein Phosphorylation and Ubiquitylation Unit, School of Life Sciences, University of Dundee, DD2 5DA, UK

*Correspondence: [p.birch@dundee.ac.uk](mailto:p.birch@dundee.ac.uk), [j.pham@dundee.ac.uk](mailto:j.pham@dundee.ac.uk)

# Supplementary Figures and Table


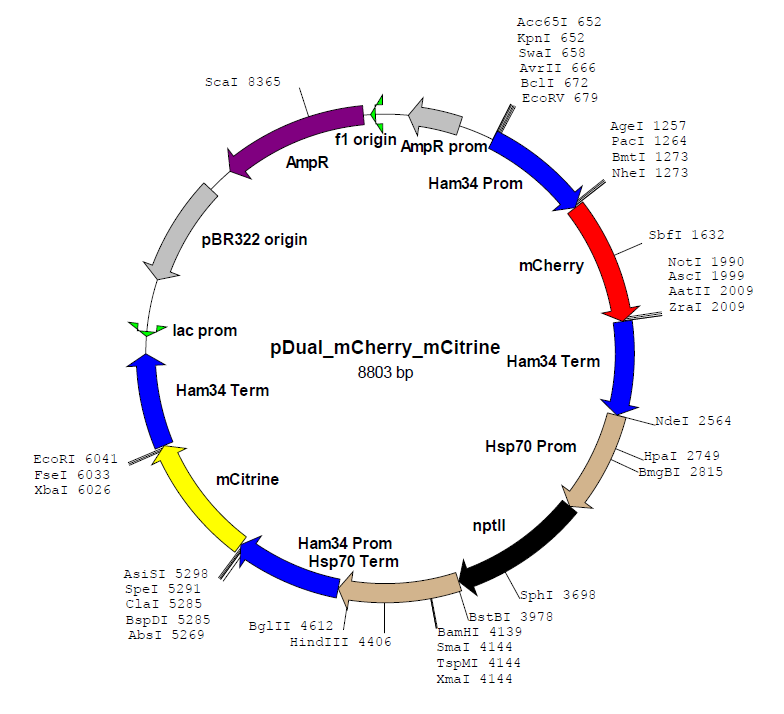


## Supplementary Fig. S1

Map of the *P. infestans* expression vector pDual_mCherry_mCitrine constructed for simultaneous expression of mCherry-tagged and mCitrine-tagged proteins.

**
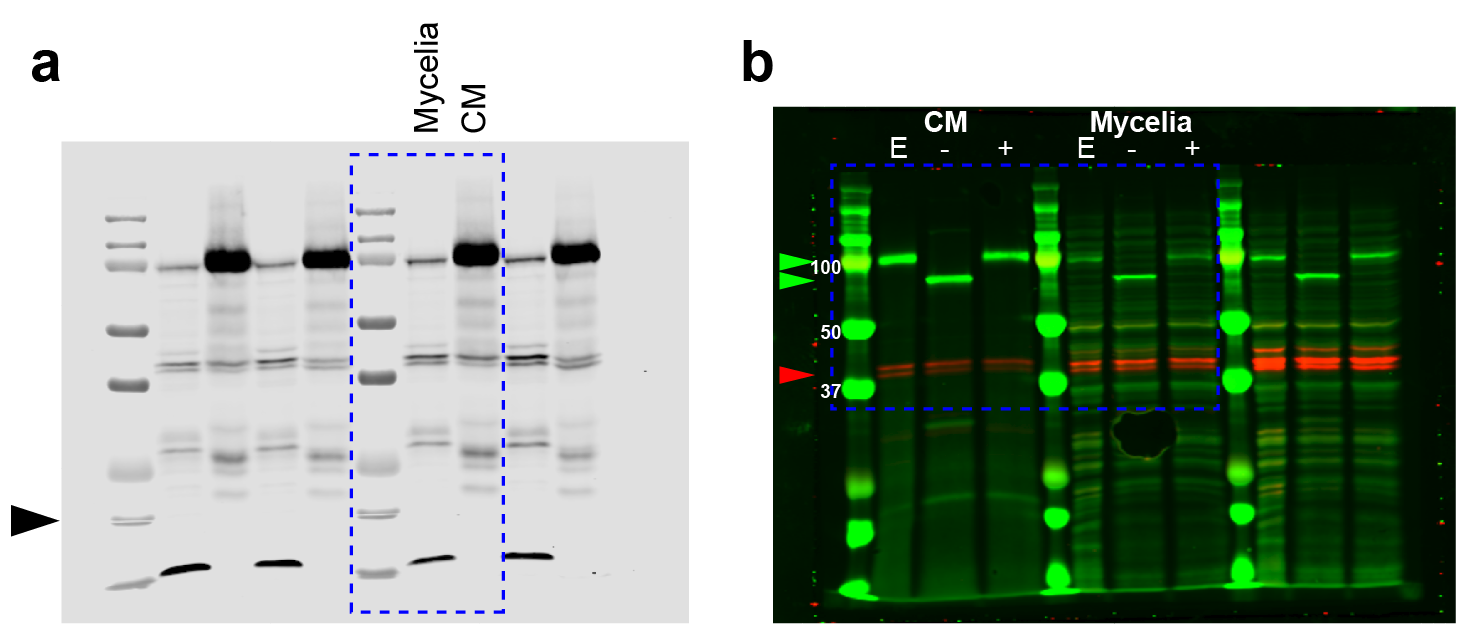
**

## Supplementary Fig. S2

**(a)** Full-length image of western blot shown in Figure 1a. The blot was cut horizontally at the position indicated () prior to hybridisation.

**(b)** Deglycosylation size shift assay for PITG_04314-mCherry () and PITG_01029-mCitrine () in *in vitro* grown mycelia and the corresponding conditioned media (CM). “E” = protein extract before treatment, “-” = treatment with no deglycosylation enzyme mix, “+” = treatment with deglycosylation enzyme mix. Size markers (kDa) are shown at left.


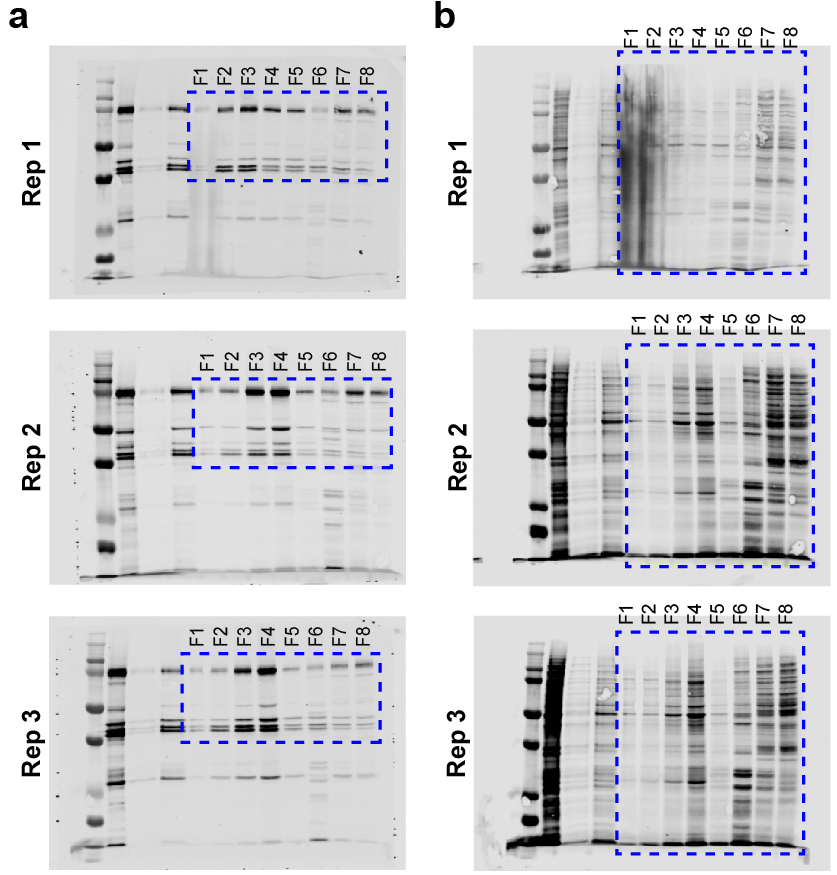


## Supplementary Fig. S3

**(a)** Full-length images of western blots shown in Figure 3a.

**(b)** Total protein stain of western blots shown in Figure 3a.


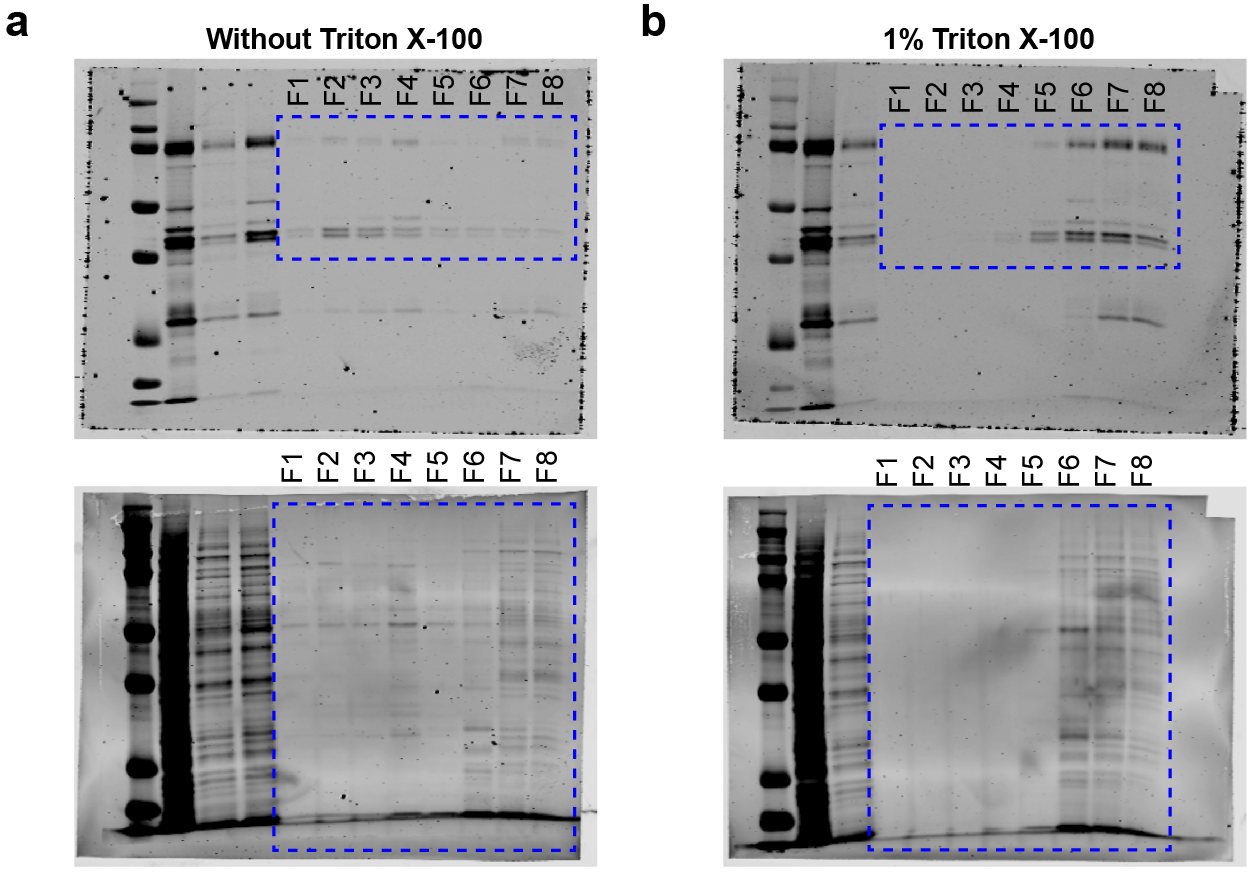


## Supplementary Fig. S4

(**a**) Full-length image (top) and total protein stain (bottom) of western blot shown in Figure 4a of fractions without Triton X-100 pre-treatment.

(**b**) Full-length image (top) and total protein stain (bottom) of western blot shown in Figure 4b of fractions with 1% Triton X-100 pre-treatment.


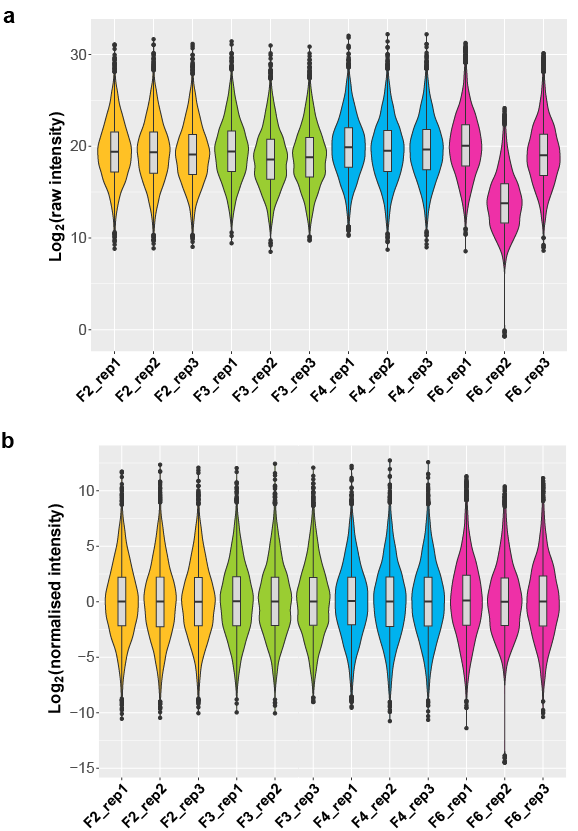


## Supplementary Fig. S5

Violin plots showing distribution of protein intensities of replicate samples within the dataset.

(**a**) distribution of Log_2_ transformed raw intensities before normalisation.

(**b**) distribution of Log_2_ transformed raw intensities after within-sample median normalisation.


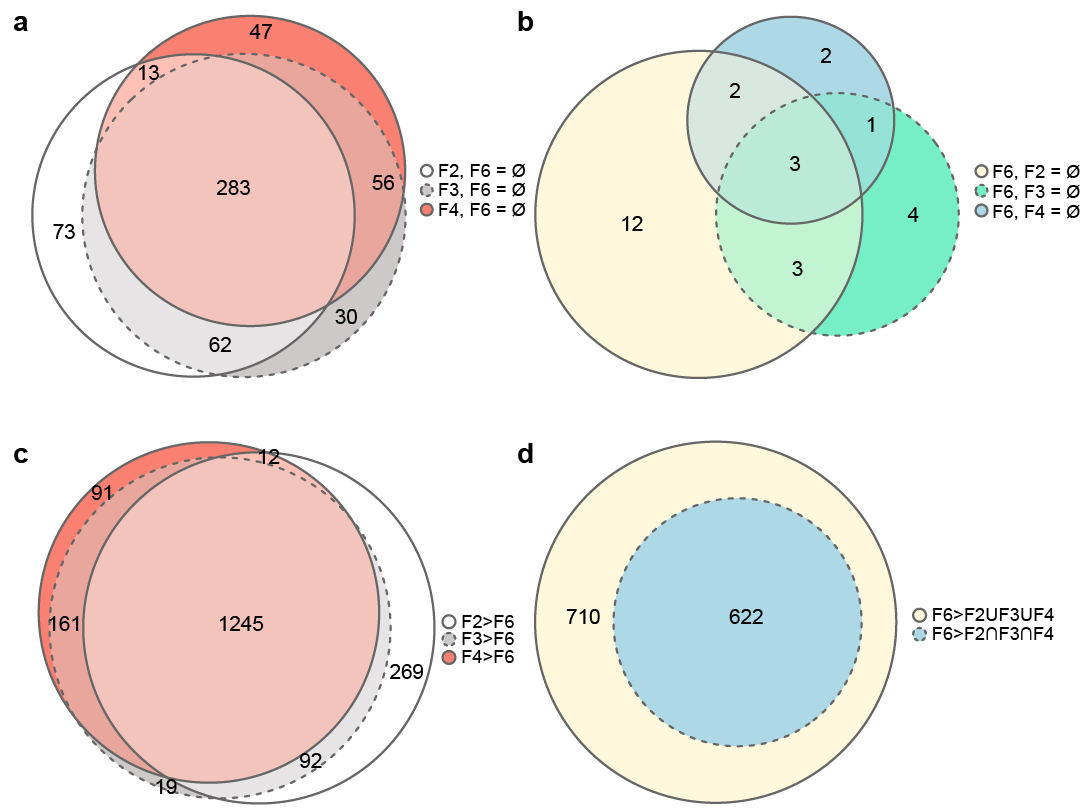


## Supplementary Fig. S6

Euler diagrams showing overlap of datasets.

(**a**) Overlap of proteins present (detected in all three biological replicates) in fractions F2, F3 and F4 whilst also absent (not detected in all three biological replicates) in F6 (F6 = Ø).

(**b**) Overlap of proteins present in F6 (detected in all three biological replicates) whilst also absent (not detected in all three biological replicates) in each buoyant fraction (F2 = Ø, 3 F3 = Ø, F4 = Ø).

(**c**) Overlap of proteins in fractions F2, F3 and F4 where Log_2_FC over F6 was greater than or equal to 0.6 and deemed statistically significant (tested by One-way ANOVA with Tukey’s HSD test (FDR <0.05 and q-value <0.05)).

(**d**) Overlap of proteins in fraction F6 where Log_2_FC of proteins found in at least one of the buoyant fractions (F2ՍF3ՍF4) over F6 and where Log_2_FC of proteins found in all buoyant fractions (F2ՈF3ՈF4) was less than or equal to -0.6. Statistical significance tested by One-way ANOVA with Tukey’s HSD test (FDR <0.05 and q-value <0.05).


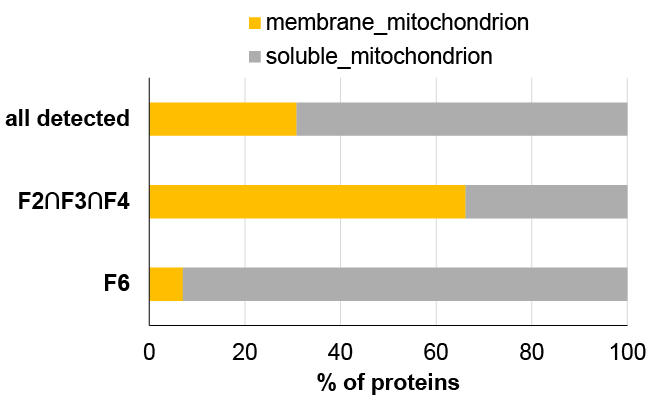


## Supplementary Fig. S7

Percentage of predicted mitochondrial membrane and soluble proteins found in the background set of all detected proteins (1,072 total), enriched in buoyant fractions (‘F2∩F3∩F4’) (319 total), and enriched in F6 (71 total).

## Supplementary Table 1

Sequences of primers used to construct vector pDual_mCherryN_mCitrine and to clone PITG_04314 and PITG_01029 into the vector for dual expression in *P. infestans.*

| **Primer** | **Sequence** | **Usage** |
| --- | --- | --- |
| ClaI_SpeI_AsiSI_mCitrine_F | 5’-AGATATCGATACTAGTTGCGATCGCACCATGGTGAGCAAGGGCGAGGA-3’ | Cloning mCitrine into pTOR |
| XbaI_FseI_EcoRI_mCitrine_R | 5’-TGAACCGCGGTTAGAATTCGGCCGGCCTTCTAGACTTGTACAGCTCGTCCATGCC-3’ |  |
| SacI_HamP_F | 5’- AGATGAGCTCGTTCCTCTCCCTCTGATGG-3’ | Cloning mCitrine flanked with HamP and HamT into pmCherryN to generate vector pDual_mCherryN_mCitrine |
| SacI_HamT_R | 5’- TGAAGAGCTCGTGGTTGGTTTACGATAAAT-3’ |  |
| AgeI_PITG_04314_F | 5’- AGATACCGGTACCATGCATTCAAGTCTTCTTTG-3’ | Cloning PITG_04314 into pDual_mCheryyN_mCitrine upstream of mCherry |
| PacI_PITG_04314_R | 5’- TGAAGAGCTCGTGGTTGGTTTACGATAAAT-3’ |  |
| ClaI_PITG_01029_F | 5’- AGATATCGATACCATGCAGATATTTGCTCCCCT-3’ | Cloning PITG_01029 into pDual_mCherryN_mCitrine upstream of mCitrine |
| AsiSI_PITG_01029_R | 5’- GGAAGCGATCGCACAGGAACGACTTGTCCACCC-3’ |  |

# Supplementary Data

## Supplementary Data 1

**a.** Dataset of all proteins detected in the study (raw intensities) before filtering for *Phaseolus vulgaris* contaminants, Log_2_ transformation, or normalisation (7,172 protein groups in total).

**b**. Dataset of all proteins detected in the study after filtering for *Phaseolus vulgaris* contaminants, Log_2_ transformation, and within sample median normalisation (6,685 protein groups in total).

**c.** High confidence proteins commonly enriched within the buoyant fractions F2, F3 and F4 (1,528 protein groups).

**d**. High confidence proteins enriched within the denser fraction F6 (625 protein groups).

## Supplementary Data 2

**a**. List of 2,335 signal peptide containing proteins combined from FungiDB, Rafaelle *et al*. ^1^ and Meijer *et al*. ^2^ against which the dataset from the present study was compared against.

**b**. List of signal peptide containing proteins (+SP) identified within the total background set of 6,685 protein groups. Those enriched within the buoyant fractions and within fraction F6 are indicated in columns ‘enriched_buoyant’ and ‘enriched_F6’, respectively.

**c**. List of verified secreted proteins published in Meijer *et al*. ^2^ identified within the total background set of 6,685 protein groups. Those enriched within the buoyant fractions and within fraction F6 are indicated in columns ‘enriched_buoyant’ and ‘enriched_F6’, respectively.

## Supplementary Data 3

**a**. ShinyGO output for Biological Processes enriched within the buoyant fractions.

**b**. Collapsed list of proteins within top three Biological Processes GO terms returned from ShinyGO for buoyant fractions with InterPro and Pfam domain descriptions.

## Supplementary Data 4

**a**. ShinyGO output for Biological Processes enriched within F6.

**b**. Collapsed list of proteins within top three Biological Processes GO terms returned from ShinyGO for F6 with InterPro and Pfam domain descriptions.

## Supplementary Data 5

**a**. List of 1,330 protein groups differentially more abundant in F2.

**b.** List of 1,909 protein groups differentially more abundant in F4.

**c**. List of 44 proteins only present in F2.

**d**. List of 75 proteins only present in F4.

**e.** ShinyGO output for Biological Processes enriched within F2.

**f**. ShinyGO output for Biological Processes enriched within F4.

# References

1 Raffaele, S., Win, J., Cano, L. M. & Kamoun, S. Analyses of genome architecture and gene expression reveal novel candidate virulence factors in the secretome of Phytophthora infestans. *BMC Genomics* **11**, 637, doi:10.1186/1471-2164-11-637 (2010).

2 Meijer, H. J. G. *et al.* Profiling the secretome and extracellular proteome of the potato late blight pathogen *Phytophthora infestans*. *Molecular & Cellular Proteomics* **13**, 2101–2113, doi:10.1074/mcp.M113.035873 (2014).
